# Succinylation enables IDE to act as a hub of larval tissue destruction and adult tissue reconstruction during insect metamorphosis

**DOI:** 10.1126/sciadv.ads0643

**Published:** 2025-02-05

**Authors:** Yan-Xue Li, Bin-Yan Shao, Ming-Ye Hou, Du-Juan Dong

**Affiliations:** Shandong Provincial Key Laboratory of Animal Cells and Developmental Biology, School of Life Sciences, Shandong University, China.

## Abstract

Metamorphosis is an important way for insects to adapt to the environment. In this process, larval tissue destruction regulated by 20-hydroxyecdysone (20E) and adult tissue reconstruction regulated by insulin-like peptides (ILPs) occur simultaneously, but the detailed mechanism is still unclear. Here, the results of succinylome, subcellular localization, and protein interaction analysis show that non-succinylated insulin-degrading enzyme (IDE) localizes in the cytoplasm, binds to insulin-like growth factor 2 (IGF-2-like), and degrades it. When the metamorphosis is initiated, 20E up-regulated carnitine palmitoyltransferase 1A (*Cpt1a*) through transcription factor Krüppel-like factor 15 (KLF15), thus increasing the level of IDE succinylation on K179. Succinylated IDE translocated from cytoplasm to nucleus, combined with ecdysone receptor to promote 20E signaling pathway, causing larval tissue destruction, while IGF-2-like was released to promote adult tissue proliferation. That is, succinylation alters subcellular localization of IDE so that it can bind to different target proteins and act as a hub of metamorphosis.

## Teaser

Succinylation alters IDE subcellular localization and interaction targets to regulate insect metamorphosis.

## INTRODUCTION

Insects are the most diverse group of organisms on earth. One of the ways for them to adapt to the environment and maintain the population is to show completely different morphologies at different stages of development, which is called metamorphosis. Metamorphosis involves not only the external morphological changes from larva to adult but also the programmed cell death (PCD) of larval tissue and the proliferation of adult tissue (*1*–*4*). How the destruction and reconstruction are carried out at the same time has received considerable critical attention in the field of development. Extensive research has shown that metamorphosis is synergistically regulated by steroid hormone 20-hydroxyecdysone (20E), peptide hormone insulin-like peptide (ILP) superfamily, and sesquiterpenoid juvenile hormone (*5*). 20E acts through the nuclear receptor complex composed of the ecdysone receptor (EcR) and ultraspiracle (USP). Then, the 20E-induced transcriptional factors such as hormone receptor 3 (HR3) and metamorphosis initiation factor Broad (Br) subsequently mediate the PCD (*6*, *7*). In addition, ILP superfamily, including insulin, insulin-like growth factor (IGF), and relaxin, promotes growth via insulin/IGF signaling (IIS) (*8*). However, the hub that enables both hormone signaling pathways to function simultaneously needs to be further identified.

Organisms can regulate physiological processes according to nutritional changes in the environment. One of the important ways is that the intermediates of nutrient metabolism act as donors of posttranslational modification (PTM) and covalently bind to functional proteins to regulate their subcellular localization, activity, stability, and protein folding in response to changes in nutrition status (*9*–*11*). Succinylation is a kind of PTM that has attracted much attention in recent years. Succinyl group can bind to ε-amino of lysine residues of target proteins in an enzymatic or nonenzymatic way. Several enzymes have been reported to be involved in the succinylation process, such as histone acetyltransferase 1 (HAT1) and carnitine palmitoyltransferase 1A (CPT1A) (*12*, *13*). Considering that the succinyl group is derived from succinic acid, an intermediate of the tricarboxylic acid cycle (TCA), succinylation may be an important mechanism for cells to regulate the process of life in response to the change of metabolic state. However, its target molecules and regulatory mechanisms still need to be further explored. The metabolism and physiological activities changed greatly during insect metamorphosis, which provides a good model for studying the mechanism of succinylation-regulating physiological process.

This study seeks to obtain data in *Helicoverpa armigera* (Lepidoptera: Noctuidae), a holometabolous agricultural pest, toexplore the role of succinylation in insect metamorphosis coordinated by 20E and ILPs. We took advantage of liquid chromatography–mass spectrometry/mass spectrometry (LC-MS/MS) to screen for the different succinylated proteins in the feeding stage and the metamorphic molting stage of the last-instar larvae. Among the differentially succinylated proteins involved in hormone regulation, we noticed a protein with important functions in both ILP and steroid hormone signaling pathway, insulin-degrading enzyme (IDE). IDE is a multifunctional zinc-metallo-endopeptidase that regulates development (*14*), which can exert protease activity to degrade insulin (*15*), glucagon (*16*), and other small molecular peptides (*17*), thereby regulating nutrient metabolism, cell proliferation, and apoptosis. It can also participate in various physiological processes through interaction with other proteins, such as regulating hormone signal pathways by binding to steroid hormone receptors (*18*). Dysfunction of IDE can lead to diabetes (*19*), Alzheimer’s disease (*17*), and other diseases. IDE has different subcellular localization in different cells, and it can be localized in the peroxisome, endosome, mitochondria, rough endoplasmic reticulum, cytoplasm, and other subcellular compartments and secreted into the extracellular space (*20*, *21*). IDE action targets are numerous and distributed in different locations of cells. However, the exact mechanism of how IDE alters subcellular localization to combine with different targets to perform different functions remains unclear. Here, we found that non-succinylated IDE localizes in the cytoplasm, where it binds to IGF-2-like and degrades it. After entering metamorphosis, 20E up-regulates the expression of carnitine palmitoyltransferase 1A (*Cpt1a*) through Krüppel-like factor 15 (KLF15) and then induces the succinylation of IDE-K179. Succinylated IDE can translocate from the cytoplasm into the nucleus and bind to the EcR-USP1 complex, promoting the 20E signaling pathway and thus promoting the PCD of larval tissue of *H. armigera*. Meanwhile, IGF-2-like was released to promote adult tissue proliferation. The results of this study revealed that succinylation regulates the subcellular localization of IDE to bind to different target proteins, participates in different signaling pathways, and actsas a hub mediating the coordination of 20E and ILPs in insect metamorphosis.

## RESULTS

### Succinylome profiling analysis identified 192 differentially succinylated proteins during metamorphosis

**Fig. 1.**
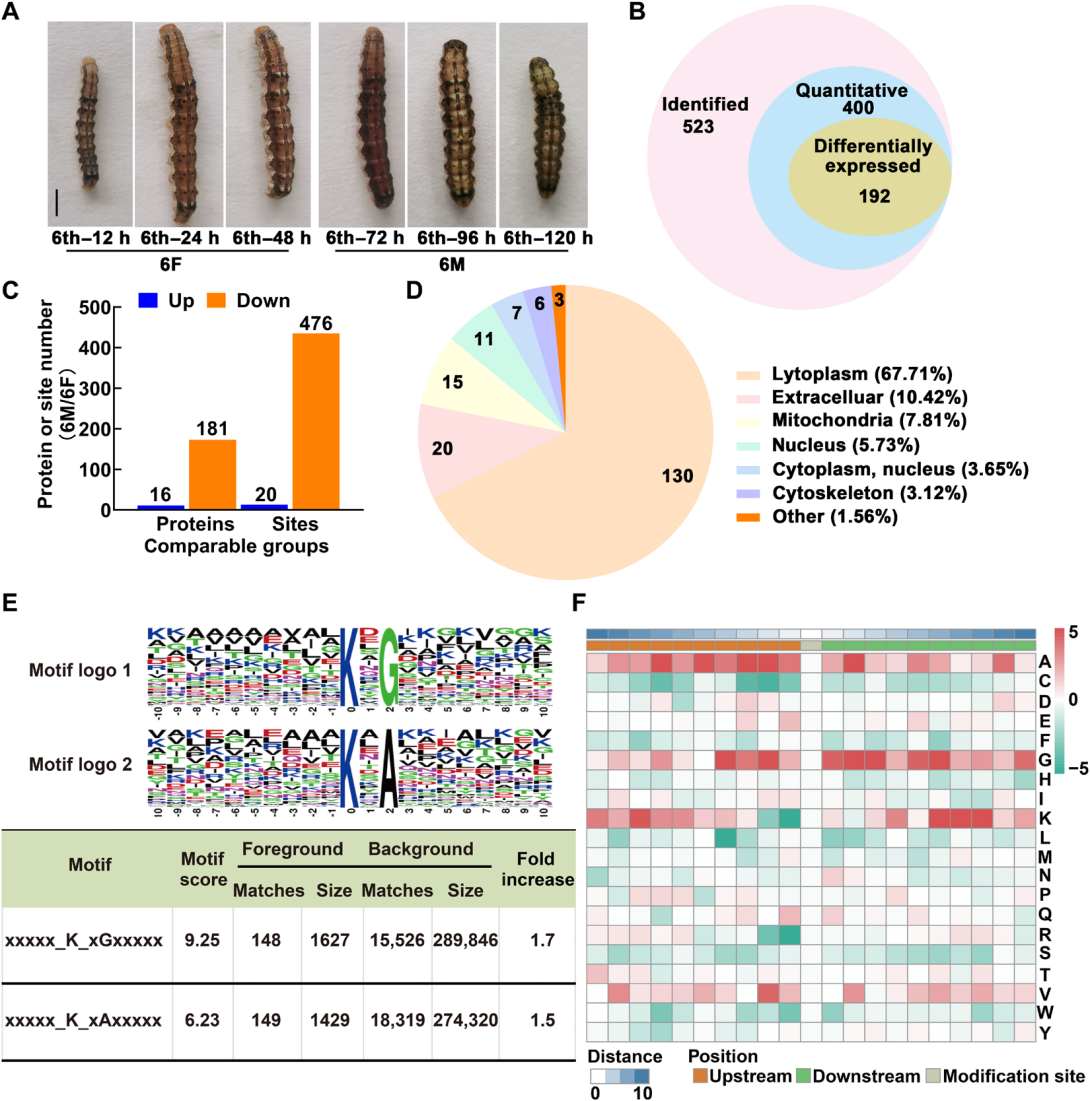
Succinylome profiling analysis identified differentially succinylated proteins during metamorphosis. (**A**) Representative images of cotton worm (*H. armigera*) during feeding and metamorphosis stages. 6F, sixth-instar feeding (12 to 60 hours after ecdysis into sixth instar) stage; 6M, metamorphic commitment stages (72 to 120 hours after ecdysis into sixth instar). Scale bar, 1 cm. (**B**) Overlap of the identified, quantified, and differentially modified succinylated proteins (fold change > 1.3). (**C**) Up-regulated and down-regulated succinylated proteins or sites during 6M compared with 6F. (**D**) Percentage of subcellular localization of differentially succinylated proteins during metamorphosis. (**E**) Motif analyses of flanking sequence preferences for succinylation sites with 1.3-fold difference, and letter size correlates to the frequency of that amino acid residue occurring in that position. (**F**) Heatmap shows the frequency of amino acids near modification sites. Red and green indicate that this amino acid is more and less abundant close to the modification site, respectively. h, hours.

The life cycle of the cotton worm consists of four stages: egg, larva, pupa, and adult. The larva stage was segmented into six instars by molts. After eating for more than 48 hours, sixth-instar larvae got into the metamorphic commitment stage at 72 hours and then pupation at about 144 hours. The whole bodies of bleeding larvae at the sixth-instar feeding (6F, 12 to 60 hours after ecdysis into sixth instar) and metamorphic commitment stages (6M, 72 to 120 hours after ecdysis into sixth instar) were collected to provide detailed illustrations of the variation of lysine succinylation in the process of metamorphosis. The sampling time point and the morphology of the larvae are shown in Fig. 1A.

Together, under the condition of false discovery rate ≤ 1%, 523 succinylation proteins were identified, of which 400 succinylation proteins were quantified and normalized to the proteome data, and 192 had a difference of more than 1.3 times between the succinylation modification states at 6F and 6M periods (Fig. 1B). Among these differential proteins, modifications at 20 succinylation sites on 16 proteins were up-regulated at the 6M stage, and modifications at 476 sites on 181 proteins were down-regulated (Fig. 1C). The detailed information of all differentially modified proteins is shown in table S1. More than half (67.71%) of the proteins were predicted to be located in the cytoplasm, 10.42% were located in the extracellular space, and 7.81% were mitochondrial proteins. In addition, they were also distributed in the nucleus and cytoskeleton (Fig. 1D). These results suggested that the succinylation status of proteins does change after the initiation of the metamorphosis program, and the degree of modification of most proteins is reduced. In addition, the differentially modified proteins were distributed everywhere in the cell, but the cytoplasmic proteins were the majority.

On the basis of the motif-x algorithm, we analyzed the sequence patterns of 10 amino acids upstream and downstream of the modified site and identified two motifs: xxxxxKsuxGxxx and xxxxxKsuxAxxx (Fig. 1E). We found that A is usually located at −9 to +6 and +9 positions, especially at −7, −5, −3, −2, and +2, and that G is usually located at −4 to +10, especially at −4, −2, +2, +3, and +6. Succinylation also occurs preferentially on peptides with K at −8, +6, +7, and +8. We also found that succinylation was least likely to occur when C was present at the −7 to −1 positions, K was present at the −1 position, L was present at the −4 position, and R was present at the −1 position (Fig. 1F).

### The differentially succinylated proteins were analyzed using GO terms and KEGG pathways

**Fig. 2.**
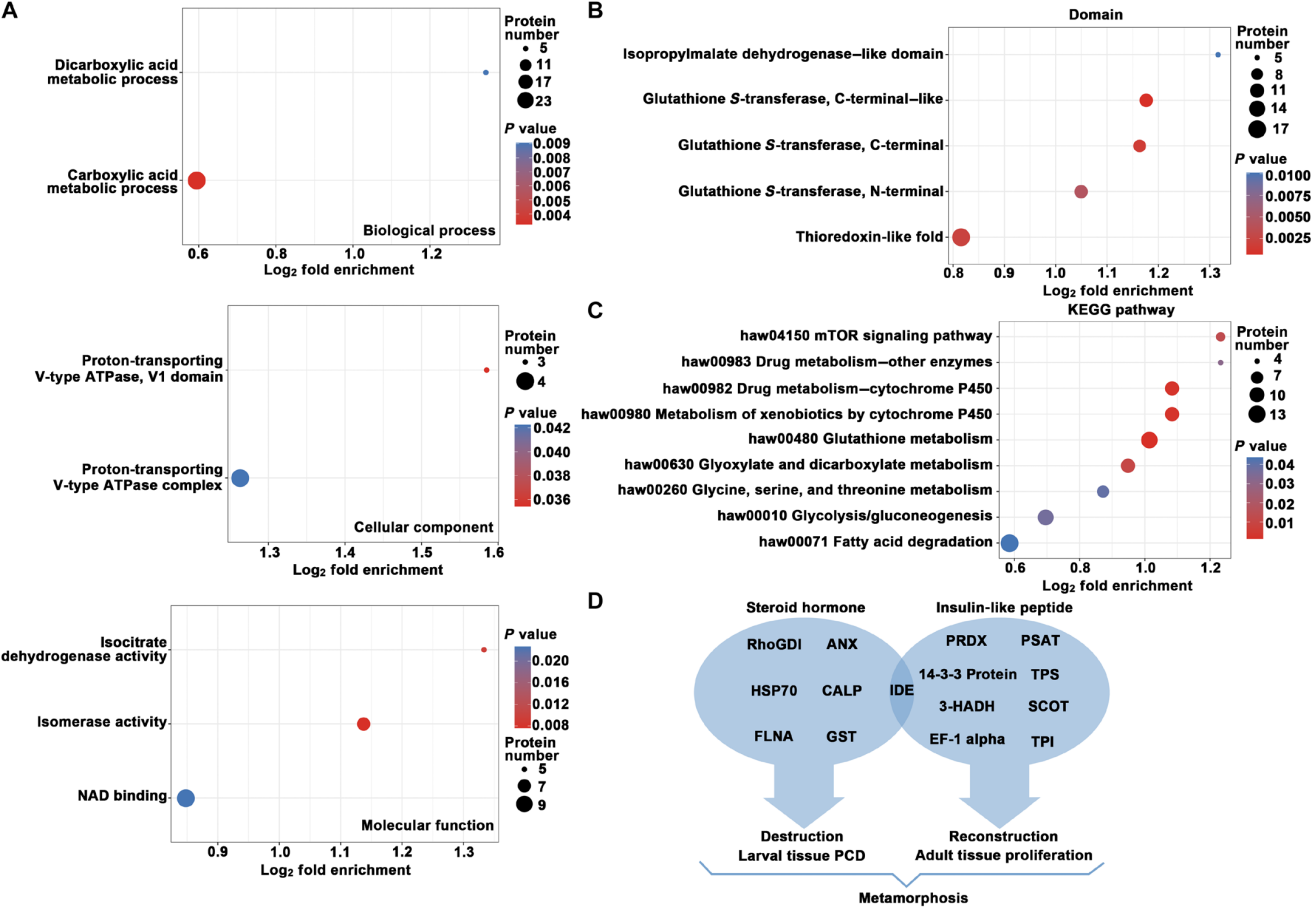
Enrichment analysis of succinylated protein during metamorphosis of *H. armigera*. (**A**) The bubble diagram shows GO-based enrichment analysis of differentially succinylated proteins. ATPase, adenosine triphosphatase; NAD, nitric acid dihydrate. (**B**) The bubble diagram shows KEGG pathway enrichment analysis of differentially succinylated proteins. (**C**) Protein domain enrichment of hypersuccinylated proteins. The vertical axis is the enrichment category, and the horizontal axis is the log_2_ transformation value of the enrichment factor. The bubble size represents the number of enriched proteins, and the bubble color represents the significance analysis. (**D**) Summary of different succinylated proteins involved in ILP or steroid hormone signaling pathways.

To further understand the biological functions of differentially succinylated proteins during metamorphosis, Gene Ontology (GO) classification and Kyoto Encyclopedia of Genes and Genomes (KEGG) enrichment analysis were performed on differentially modified proteins. The biological effects of proteins were explained from different perspectives such as biological process, molecular function, and metabolic pathway, and the results were shown with a bubble diagram (Fig. 2). The bubble plot shows that, in terms of biological processes, carboxylic and dicarboxylic acid metabolism was significantly enriched. In terms of cellular component, proton-transporting V-type adenosine triphosphatase (ATPase) was enriched, and, in terms of molecular functions, isocitrate dehydrogenase activity, isomerase activity, and nitric acid dihydrate binding activity were mainly enriched (Fig. 2A). Protein domain enrichment analysis also showed that many redox-related protein domains were enriched (Fig. 2B). KEGG pathway enrichment analysis showed that succinylated differentially modified proteins were most enriched in pathways such as glutathione metabolism, cytochrome P450–involved drug metabolism, glyoxylate and dicarboxylate metabolism, and the mammalian target of rapamycin (mTOR) signaling pathway. In addition, the nutrient metabolic pathways such as fatty acid degradation, glycolysis/gluconeogenesis, and glycine, serine, and threonine metabolism were also significantly enriched (Fig. 2C). The differentially succinylated proteins involved in the nutrient metabolic pathways are shown in detail in fig. S1.

The specific objective of this study was to explore which of these differentially modified proteins are involved in hormone regulation. Figure 2D shows an overview of differentially succinylated proteins that may be involved in ILP or steroid hormone signaling pathways. The detailed information is shown in table S2 (*22*–*36*). What stands out in Fig. 2D is that IDE appears at the intersection of two hormonal pathways. We performed functional enrichment analyses of the up-regulated succinylated proteins on the basis of GO, and the results were shown with column diagram (fig. S2A). Furthermore, IDE was distributed in both cytoplasm and nucleus (fig. S2B). So, whether succinylation alters the IDE’s subcellular localization and target protein selection so that it participates in different hormone signaling pathways and thus in the regulation of insect metamorphosis is what we want to study.

### The IDE is succinylated and localized in the nucleus during metamorphosis

To further confirm the results of succinylome analysis, we identify the IDE in the genome of *H. armigera* (GenBank, accession number ON146199) and prepare polyclonal antibodies to IDE. We first performed a phylogenetic tree analysis. The phylogenetic tree showed that the lepidopterous insects—*H. armigera*, *Spodoptera litura*, and *Bombyx mori*—were clustered in one group (fig. S3A). IDE domain prediction of *H. armigera*, *Homo sapiens*, and *B. mori* showed that their IDE domains were very similar, containing two typical insulinase domains: Peptidase_M16 and Peptidase_M16_C superfamily domains, where Peptidase_M16 is the functional domain of insulinase. Peptidase_M16_C, an inactive domain of insulinase, may play a role in the targeted substrate (fig. S3B).

The specificity of the antibody was detected by sixth-instar 96-hour (6th–96-h) fat-body samples. Western blot results showed that the antibody recognized the IDE with a molecular weight of 116 kDa in the tissue with a single band (fig. S4), indicating a reasonable antibody specificity and could be used in subsequent experiments.

**Fig. 3.**
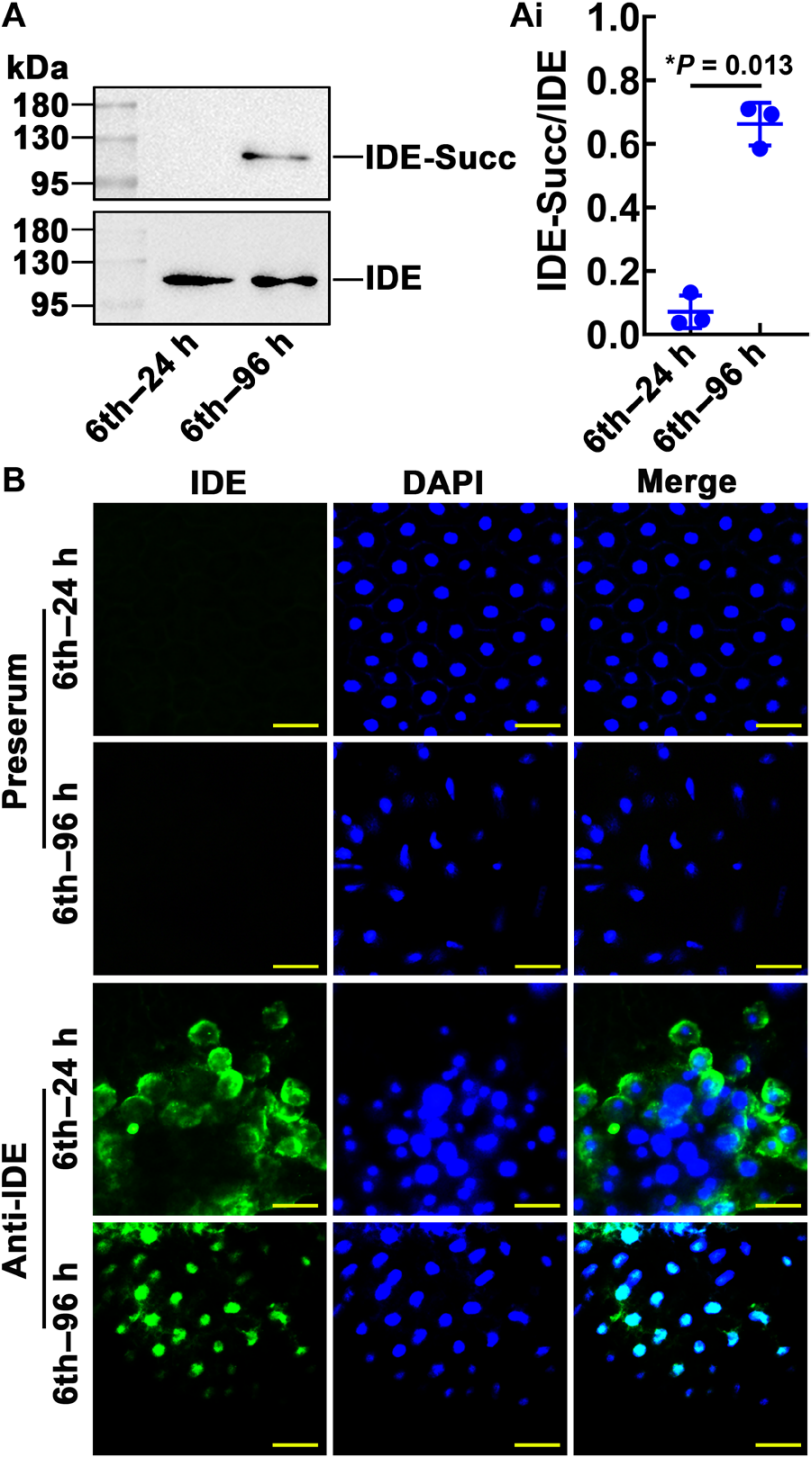
Expression profile of succinylated IDE and IDE location. (**A**) The variation of IDE succinylation levels in the fat body during larval development was detected by antibodies against succinylation. IDE-Succ was the succinylated form of IDE. The amount of IDE was affinity enriched using the antibody of IDE. [(A), i] Quantitative analysis of the IDE succinylation using ImageJ software. (**B**) IDE was localized in the fat body. Green fluorescence: IDE detected by anti-IDE rabbit polyclonal antibodies and Alexa Fluor 488–conjugated goat antirabbit IgG antibodies. Blue fluorescence: Nuclei stained with 4′,6-diamidino-2-phenylindole (DAPI) dihydrochloride. Preserum was used as IDE antibody control. Merge: Merging of different fluorescent signals. Scale bars, 50 μm. The error bar indicates the means ± SD of three biological replicates. The Student’s *t* test was used to show significant differences (*P < 0.05 and **P < 0.01). h, hours.

The PTM profiles were verified by immunoblotting. The succinylated IDE in the fat body was detected in the 6th–96-hour larvae during metamorphosis (Fig. 3, A and Ai). This observation was consistent with the results of omics. Immunohistochemistry showed that IDE was mainly localized in the cytoplasm of the fat-body cell of 6th–24-hour larvae and the nucleus of fat-body cell of 6th–96-hour larvae. The negative control preserum did not detect IDE in the fat body of 6th–24-hour and 6th–96-hour larvae (Fig. 3B). These results suggested that succinylation of IDE and IDE’s nuclear localization may play a vital role during metamorphosis.

### 20E induced IDE succinylation at K179, leading to IDE translocation into the nucleus

**Fig. 4.**
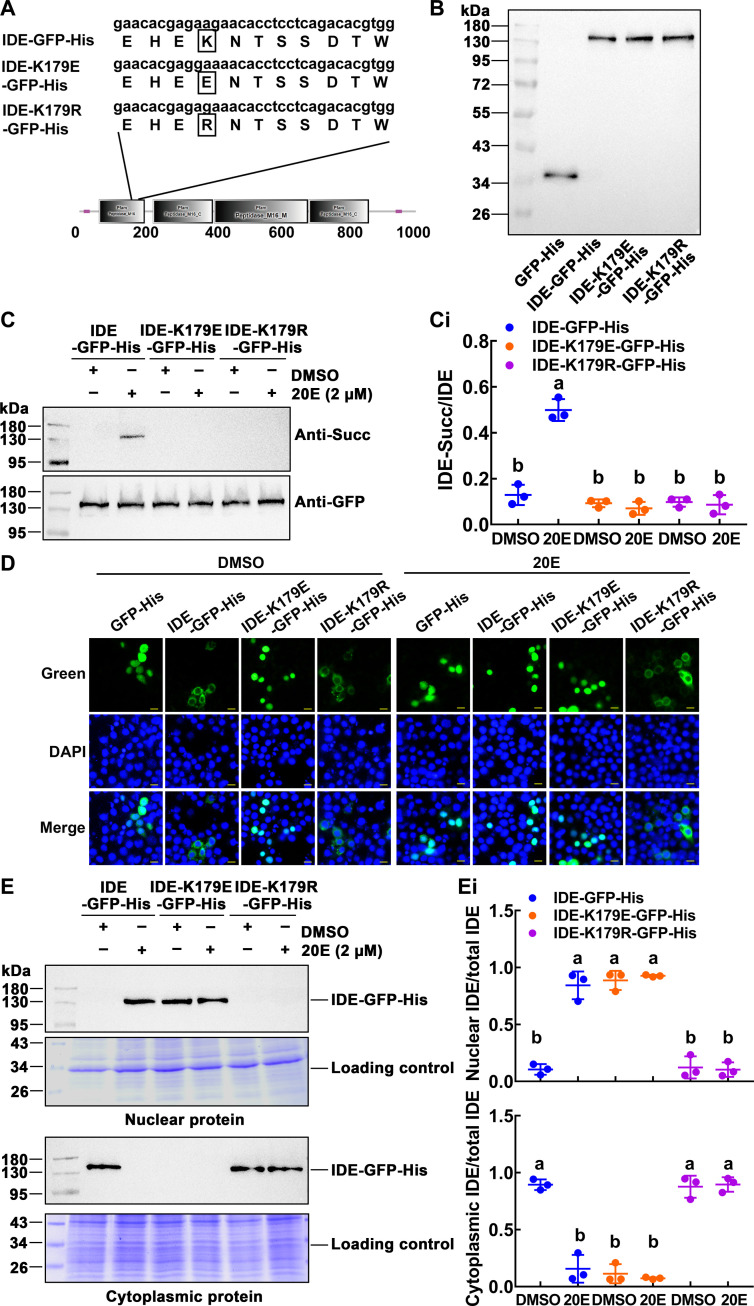
20E induced the succinylation of IDE and nuclear localization. (**A**) The diagram of the mutant plasmid. The IDE succinylation modification sites predicted by the succinylation proteomics, IDE-K179E-GFP-His– and IDE-K179R-GFP-His–mutated plasmids, were designed to simulate succinylation and desuccinylation. (**B**) Western blotting detection of overexpressed IDE and IDE mutants in HaEpi cell lines. The target protein was detected by antibody against GFP. (**C**) Changes of IDE succinylation by 20E stimulation. Cells were incubated in 2 μM 20E for 1 hour. [(C), i] Statistical analysis of IDE succinylation in (C). (**D**) The subcellular localization of IDE-GFP-His, IDE-K179E-GFP-His, and IDE-K179R-GFP-His in the cells. The cell was induced by 2 μM 20E for 1 hour. Green: Green fluorescence protein. DAPI: Nuclei stained as blue fluorescence. Scale bars, 20 μm. (**E**) Western blotting demonstrated the subcellular distribution of IDE-GFP-His and mutants. Coomassie Brilliant Blue staining was used as a loading control for nuclear or cytoplasmic protein quantity and quality. [(E), i] Quantitative analysis of the distribution of IDE-GFP-His and mutants in nucleus and cytoplasm. ImageJ was used to calculate the value of the band’s gray level in (E). All the experiments were repeated in triplicate, and statistical analysis was conducted using analysis of variance (ANOVA). The different lowercase letters show significant differences (*P <* 0.05). The bars indicate means ± SD.

To explore the role of IDE succinylation in metamorphosis development, we first need to identify the sites where succinylation occurs. Mass spectrometry predicted K179 as modified site. IDE–green fluorescent protein (GFP)–His and two mutant plasmids, IDE-K179E-GFP-His and IDE-K179R-GFP-His, were constructed (Fig. 4A). The IDE-K179E-GFP-His was constructed to mutate lysine residue into negatively charged glutamate residue to mimic succinylation and IDE-K179R-GFP-His plasmid to mutate lysine residue into positively charged arginine residue to mimic desuccinylation (*37*). Western blotting showed the expression of wild-type and mutant IDE, with GFP-His as a tag control (Fig. 4B).

Considering that the mass spectrometry results showed that IDE succinylation levels increased during the metamorphosis with higher 20E titers, we treated the HaEpi cells with dimethyl sulfoxide (DMSO) and 20E, respectively. As can be seen in Fig. 4 (C and Ci), the wild-type IDE-GFP-His showed an increase of succinylation levels under 20E induction. In contrast, the succinylation of the IDE-K179E-GFP-His and IDE-K179R-GFP-His mutant was not detected under 20E induction, suggesting that K179 is the site of succinylation modification and that the succinylation is induced by 20E.

To further explore the relationship between succinylation and nuclear localization of IDE, the subcellular location of IDE was analyzed by immunocytochemistry. IDE-GFP-His was translocated into the nucleus after 20E treatment for 1 hour, compared with that after DMSO treatment. IDE-K179E-GFP-His appeared in the nucleus under both DMSO and 20E treatment; however, IDE-K179R-GFP-His remained in the cytoplasm under both DMSO and 20E treatment, and GFP as tag control was detected throughout the cell (Fig. 4D). Western blotting confirmed that IDE-GFP-His was translocated into the nucleus by 20E induction; IDE-K179E-GFP-His was always in the nucleus; however, IDE-K179R-GFP-His was always in the cytoplasm (Fig. 4, E and Ei). These data suggested that 20E induces IDE-K179 succinylation and nuclear translocation.

### The succinylated IDE interacted with EcR to promote *Hr3* transcription

**Fig. 5.**
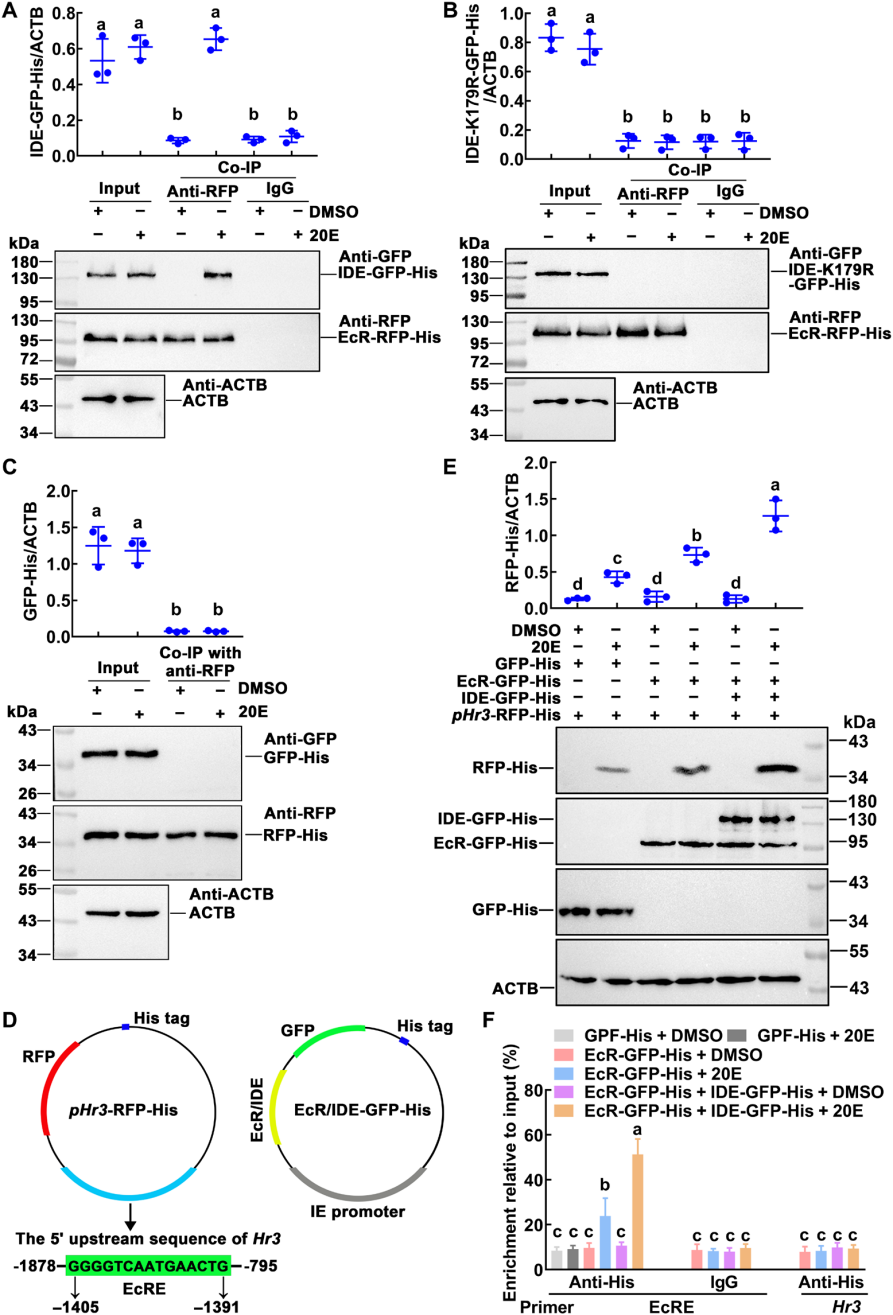
20E induced the interaction of IDE with EcR to promote the expression of *Hr3*. (**A** and **B**) IDE-GFP-His coupling with EcR-RFP-His under 20E induction (2 μM for 1 hour). DMSO was used as solvent control. Input: The levels of IDE-GFP-His, IDE-K179R-GFP-His, and EcR-RFP-His in the cells detected by an antibody against GFP or RFP. ACTB was a loading control. Co-IP: Anti-RFP antibody co-immunoprecipitated EcR-RFP-His. Nonspecific mouse IgG was a negative control. Ten percent gel in SDS–polyacrylamide gel electrophoresis (SDS-PAGE). (**C**) Co-immunoprecipitation (Co-IP) detected the interaction of GFP-His and RFP-His. Input: The levels of GFP-His and RFP-His in the cells detected by an antibody against GFP or RFP. ACTB was a loading control. Co-IP: Anti-RFP antibody co-immunoprecipitated RFP-His. Ten percent gel in SDS-PAGE. (**D**) The report plasmid diagram of *pHr3*-RFP-His. (**E**) Western blotting showed the expression of the *pHr3*-RFP-His reporter plasmid (RFP-His) when EcR-GFP-His, IDE-GFP-His, or GFP-His was overexpressed under 20E or DMSO treatment. ACTB was a loading control. (**F**) Chromatin immunoprecipitation (ChIP) assays confirmed that overexpressed IDE-GFP-His enriched more fragments of EcRE motif under 20E induction. The primers EcRE are the sequences containing EcRE in the *Hr3* promoter region. Primer *Hr3* targeting the *Hr3* coding DNA sequence (CDS) was used as a control. The data were performed by analysis of ANOVA. The different lowercase letters show significant differences (*P* < 0.05). Data are the means ± SD of three replicates.

What is the role of succinylation of IDE into the nucleus during metamorphosis? Previous studies on IDE have revealed the interaction of IDE with androgen receptor (AR) and glucocorticoid receptor (GR) (*18*). Considering that androgens and glucocorticoids are both steroid hormones, we examined the association between IDE and EcR, the receptor of 20E, a steroid hormone that regulates metamorphosis. Co-immunoprecipitation (Co-IP) analysis showed that the EcR–red fluorescent protein (RFP)–His could be co-precipitated together with IDE-GFP-His under 20E induction, when EcR-RFP-His and IDE-GFP-His were co-overexpressed in the HaEpi cells (Fig. 5A). However, EcR-RFP-His could not co-precipitated with IDE-K179R-GFP-His (Fig. 5B). GFP-His and RFP-His were co-overexpressed in the cells as tag controls, and their interaction was not detected (Fig. 5C). These results indicated that 20E induces a physical interaction between succinylated IDE and EcR.

To further address the outcome of IDE succinylation in the 20E pathway, we examined the expression of *Hr3*, a key transcription factor of 20E signaling pathway. The *pHr3*-RFP-His reporter plasmid (*38*) was used to analyze the change of *Hr3* transcription levels when IDE was overexpressed in the cells (Fig. 5D). Western blotting showed that, when IDE-GFP-His was overexpressed, the level of the reporter protein RFP-His was significantly increased under 20E treatment compared with that of the control group (Fig. 5E). We detected the binding of EcR-RFP-His to EcRE using a chromatin immunoprecipitation (ChIP) assay. Using the primer of EcRE showed that EcR-RFP-His enriched more EcRE-containing fragments when IDE-GFP-His was overexpressed and exposed to 20E compared with that of other controls. Using the primer of *Hr3* that amplifies the coding region of *Hr3* as a nonspecific binding control, no EcRE-containing fragment was enriched under 20E or DMSO treatment (Fig. 5F), suggesting that succinylated IDE promotes EcR binding to the EcRE for *Hr3* expression.

### Non-succinylated IDE resulted in reduced IGF-2-like levels

**Fig. 6.**
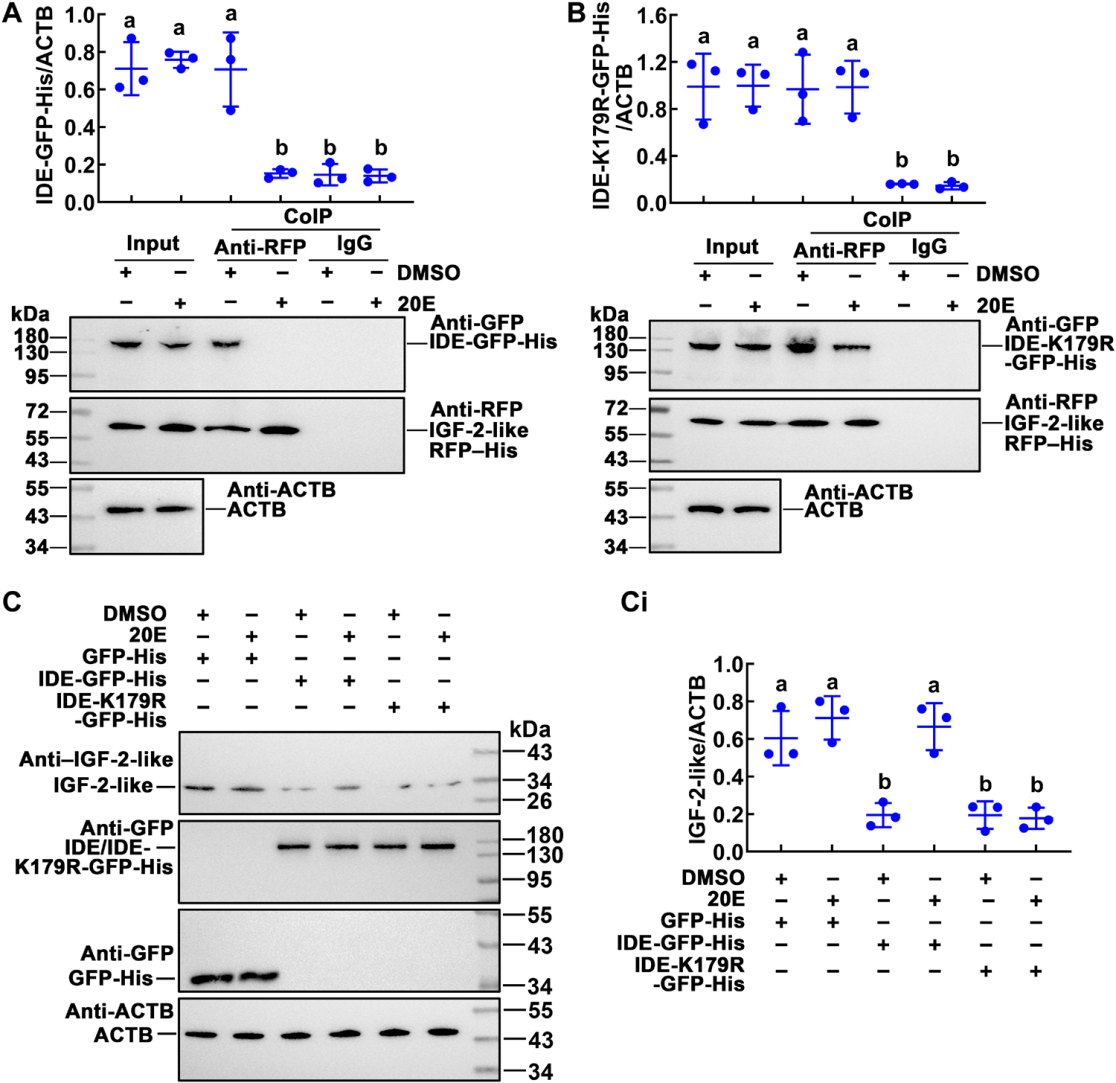
Non-succinylated IDE interacted with IGF-2-like to reduce the IGF-2-like levels. (**A** and **B**) IDE-GFP-His interacted with IGF-2-like–RFP–His under DMSO treatment. IDE-K179R-GFP-His interacted with IGF-2-like–RFP–His under 20E (2 μM for 1 hour) or DMSO treatment. Input: The levels of IDE-GFP-His, IDE-K179R-GFP-His, and IGF-2-like–RFP–His in the cells detected by an antibody against GFP or RFP. ACTB was a loading control. Co-IP: Anti-RFP antibody co-immunoprecipitated IGF-2-like–RFP–His. Nonspecific mouse IgG was a negative control. Ten percent gel in SDS-PAGE. (**C**) Western blotting analysis of the IGF-2-like level, after overexpression of IDE-GFP-His, IDE-K179R-GFP-His, or GFP-His. Cells were incubated in 2 μM 20E for 1 hour, and DMSO was used as solvent control. [(C), i] Quantification of the data in (C) using ImageJ software. Statistical analysis was performed using three independent replicates by ANOVA; different letters represented significant differences (*P* < 0.05). The bars indicate the means ± SD of three replicates.

IDE has the ability to degrade a variety of small-molecule peptides, and ILP superfamily is one of its targets. To investigate the regulation of PTMs to the function of IDE, we examined the association between IDE and IGF-2-like, a member of the ILP superfamily, which promotes insect metamorphosis (*39*). Co-IP assay using the antibody against GFP (anti-GFP) confirmed that the overexpressed IGF-2-like–RFP–His protein interacted with IDE-GFP-His under DMSO treatment (Fig. 6A). When IDE-K179R-GFP-His and IGF-2-like–RFP–His were co-overexpressed in the cells, co-IP results showed that IDE-K179R-GFP-His and IGF-2-like–RFP–His existed in complex form under DMSO or 20E treatment (Fig. 6B). Therefore, we performed an experiment in which IDE-GFP-His or IDE-K179R-GFP-His was overexpressed and analyzed the IGF-2-like levels. The IGF-2-like levels were significantly reduced in the cells that overexpressed IDE-GFP-His under DMSO treatment or overexpressed IDE-K179R-GFP-His under DMSO or 20E treatment (Fig. 6, C and Ci). These results suggested that the non-succinylated IDE can degrade IGF-2-like.

### Succinylation of IDE-K179 promoted gene expression of the 20E signaling pathway

**Fig. 7.**
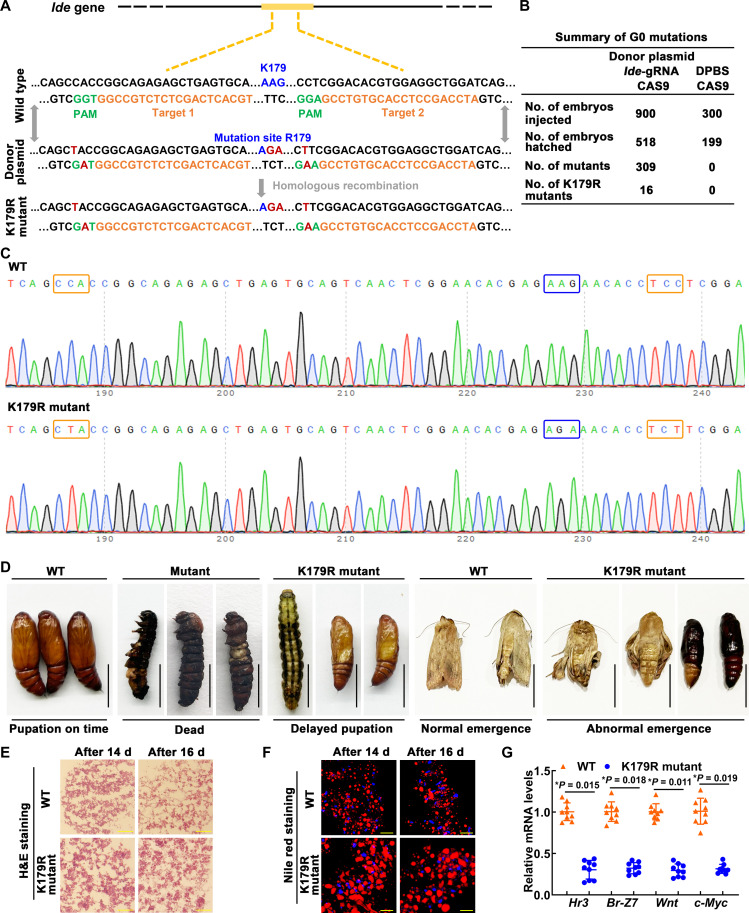
The roles of IDE in metamorphosis were determined by CRISPR-Cas9 system. (**A**) Schematic showing CRISPR-Cas9 system–mediated mutants. The black line refers to the genome of *H. armigera*; the yellow block represents the exon. Orange sequence, an ~20-nucleotide guide sequence; green sequence, 5′-NGG-3′ adjacent motif (PAM); blue sequence, wild sequence; red sequence, mutant sequence. (**B**) Summary of G0 generation. (**C**) Mutation sequence was identified by TA cloning and Sanger sequencing. The PAMs were marked with an orange box. The mutation site was marked with a blue box. (**D**) Images demonstrating WT and mutant *H. armigera* phenotypes. Scale bars, 1 cm. (**E**) The fat-body morphology was assessed by H&E staining. Scale bars, 50 μm. (**F**) The lipid droplet (LD) changes of the fat body were assessed by Nile red staining. Scale bars, 50 μm. d, days. (**G**) qRT-PCR analysis of the mRNA levels of genes in WT and mutant *H. armigera*. Statistical analysis was performed using Student’s *t* test. The bars indicate the means ± SD of three replicates.

To confirm the effect of succinylation of IDE-K179 on the metamorphosis of *H. armigera*, we introduced the K179R mutation using a CRISPR-Cas9 system. We introduced the K179R mutation using guide RNA (gRNA) at both ends of the target site and donor plasmid containing the target site mutation (Fig. 7A). In the control group, 300 eggs were injected with Dulbecco’s phosphate-buffered saline (DPBS) and Cas9 protein, and 199 embryos hatched, giving a hatching rate of 66.3%. Meanwhile, in the experimental group, 900 eggs were injected with two gRNA, Cas9 protein and donor plasmid, and 518 embryos hatched, giving a hatching rate of 57.6% (Fig. 7B). Genotypic analysis showed that mutations were found in 62.7% of the incubated larvae, including 309 larvae (mutant, 59.7%) with multiple destruction of IDE from 165 to 189 amino acids and 16 larvae (K179R mutant, 3%) with only editing of PAM and K179 sites (Fig. 7B and fig. S5). Chromatographic analysis showed the K179R mutation in the heterozygous mutant (Fig. 7C). The mutant group survived to the sixth instar and died at the end of the sixth instar. The K179R mutant group has delayed development but can survive to the pupal stage and lastly show abnormal emergence, including failure to develop the wings properly and death in the pupal stage (Fig. 7D). Further, we analyzed the cause of the abnormal emergence of the K179R mutant group. Hematoxylin and eosin (H&E) staining showed that the fat body of K179R mutant larvae was still closely arranged on the 14th and 16th days after hatching from the egg (about sixth instar, 96 and 144 hours, prepupae stage); however, the WT control larvae showed the fat-body dissociation (Fig. 7E). Nile red staining showed that the K179R mutant larval lipid drops were kept large and dense, while the lipid drops of control larvae became smaller (Fig. 7F). These phenotypes indicate that the metamorphosis of the K179R mutant group is inhibited, so we also analyzed the transcription of closely related genes, including *Hr3* and *Br-Z7* in the 20E signaling pathway and *Wnt* and *c-Myc* in the proliferation-related pathway. We found decreased *Hr3*, *Br-Z7*, *c-Myc*, and *Wnt* expression in K179R mutants (Fig. 7G). These results indicated that succinylation of IDE-K179 plays an important role in enhancing 20E signaling and promoting imaginal tissue growth and proliferation.

**Fig. 8.**
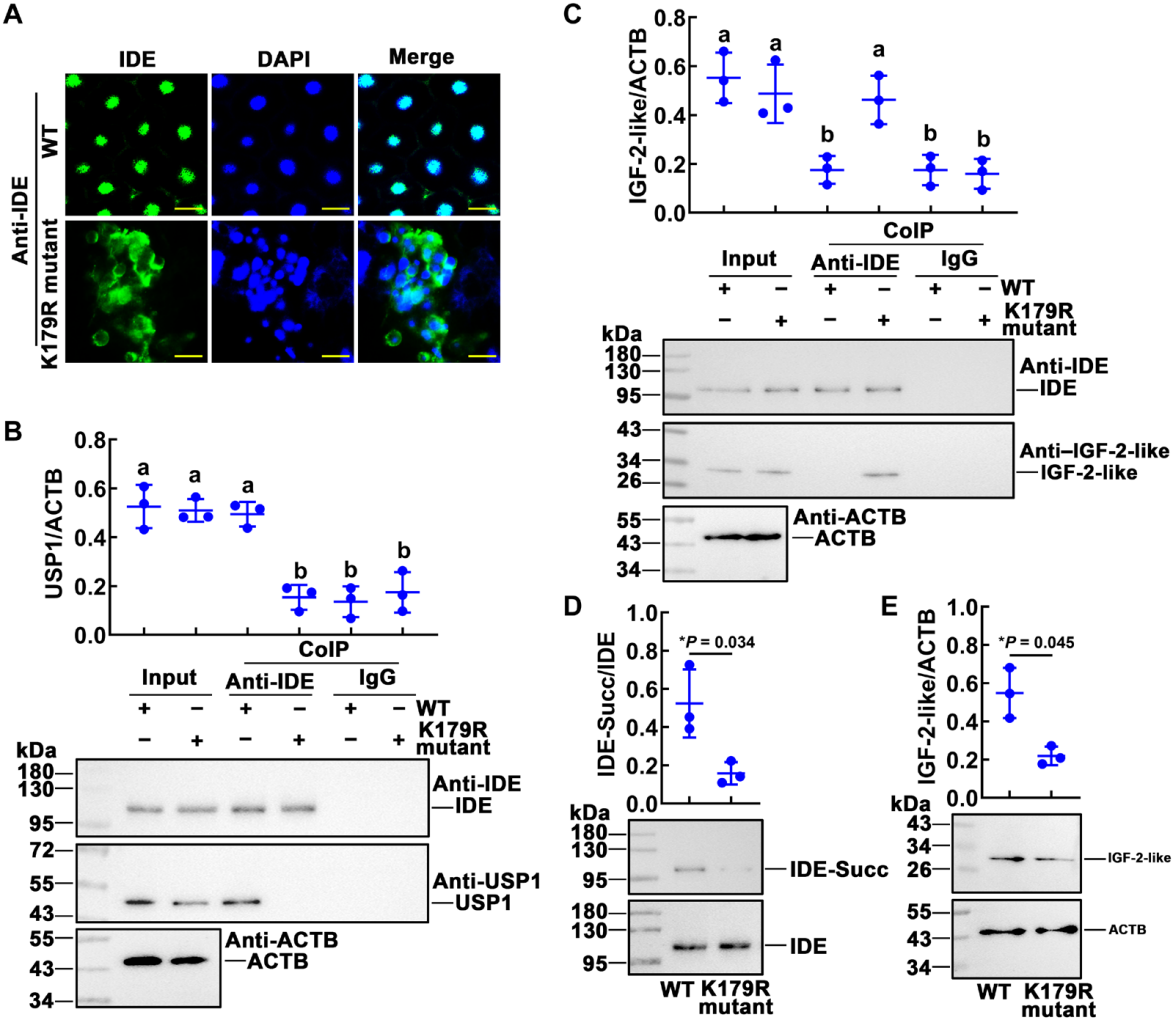
K179 mutation blocked the 20E signaling pathway. (**A**) IDE localization in the fat body of wild-type and K179R mutant larvae by immunohistochemical analysis. Green fluorescence indicates IDE, and nuclei were stained with DAPI (blue). Scale bars, 20 μm. (**B**) Co-IP detected the interaction of IDE and USP1. Input: The levels of IDE and USP1 in the fat body detected by an antibody against IDE or USP1. ACTB was a loading control. Co-IP: Anti-IDE antibody co-immunoprecipitated IDE. Nonspecific rabbit IgG was a negative control. Ten percent gel in SDS-PAGE. (**C**) K179R-IDE interacted with IGF-2-like. Input: The levels of IDE and IGF-2-like in the fat body detected by an antibody against IDE or IGF-2-like. ACTB was a loading control. Co-IP: Anti-IDE antibody co-immunoprecipitated IDE. Nonspecific rabbit IgG was a negative control. Ten percent gel in SDS-PAGE. (**D**) The levels of succinylated IDE in the fat body of WT and K179R mutant. IDE-Succ was the succinylated form of IDE. The amount of IDE was affinity enriched using the antibody of IDE. (**E**) Western blotting analysis of the IGF-2-like levels in the fat body of WT and K179R mutant. Statistical analysis was performed using ANOVA or Student’s *t* test; different letters represented significant differences (*P* < 0.05). The bars indicate the means ± SD of three replicates.

We further analyzed the mechanism of IDE involvement in the metamorphosis development of *H. armigera* using fat-body samples of larvae hatched from eggs on the 14th day. Immunohistochemistry showed that the IDE of the wild type was mainly located in the nucleus and that of the K179R mutant was mainly located in the cytoplasm (Fig. 8A). From the results of Co-IP, we can see that the wild-type IDE can form complexes with USP1, while the IDE K179R mutant interacts with IGF-2-like (Fig. 8, B and C). Mutations at the IDE-K179R site resulted in significant reductions in IDE succinylation and IGF-2-like levels (Fig. 8, D and E). These results suggested that succinylated IDE is located in the nucleus and forms complexes with USP1 to promote 20E signaling pathway gene expression, while non-succinylated IDE binds to IGF-2-like to promote its degradation.

### 20E promoted succinylation of IDE by up-regulating *Cpt1a* expression

**Fig. 9.**
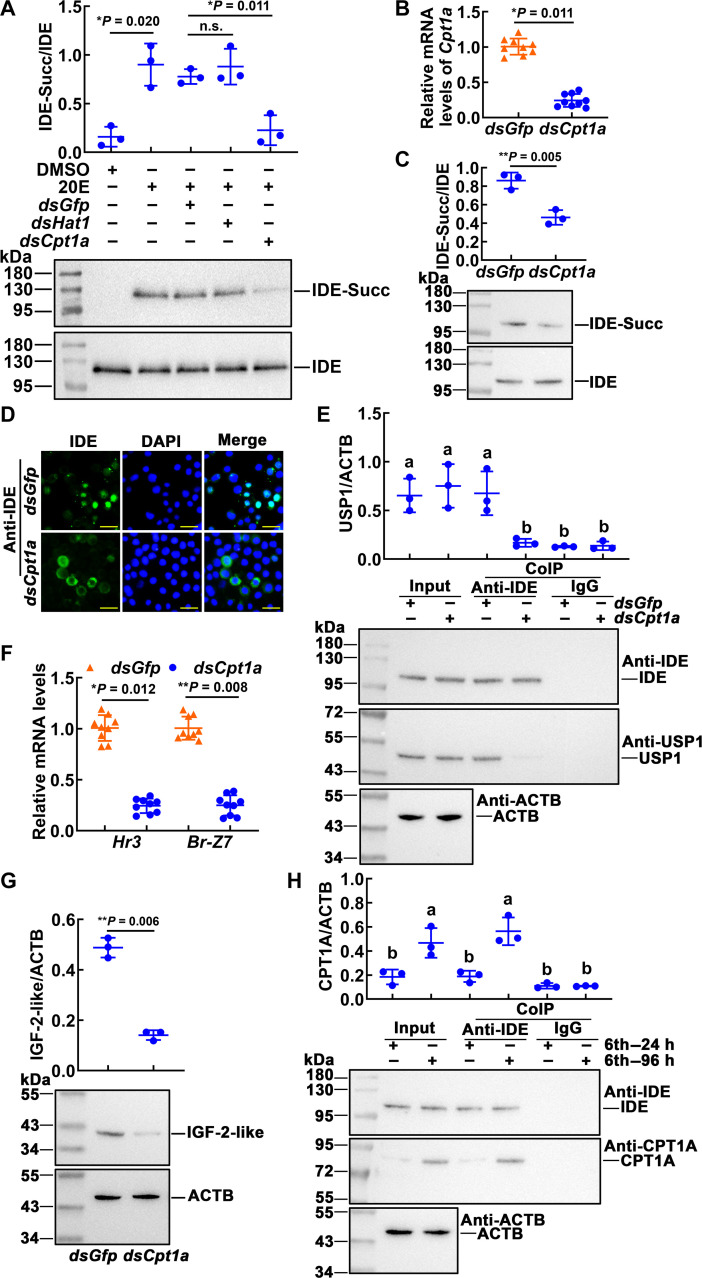
CPT1A regulated the succinylation level of IDE. (**A**) *Cpt1a* involved in 20E-induced succinylation of IDE. The cells were transfected with 2 μg of dsRNA for 48 hours. 20E (2 μM) was added to the cells for 1 hour. *dsGfp* was used as the control. (**B**) The expression of *Cpt1a* in the fat body of dsRNA (1 μg per larva, 24 intervals, four times) injection. (**C**) Western blotting analysis of IDE succinylation levels in the fat body. The amount of IDE was affinity enriched using the antibody of IDE. (**D**) IDE localization in the fat body of dsRNA injection by immunohistochemical analysis. Green fluorescence indicates IDE, and nuclei were stained with DAPI (blue). Scale bars, 20 μm. Samples were taken 96 hours after the first dsRNA injection. (**E**) Co-IP detected the interaction of IDE and USP1. Input: The levels of IDE and USP1 in the fat body detected by an antibody against IDE or USP1. ACTB was a loading control. Co-IP: Anti-IDE antibody co-immunoprecipitated IDE. Nonspecific rabbit IgG was used as a negative control. Ten percent gel in SDS-PAGE. (**F**) The expression of *Hr3 and Br-Z7* after knockdown of *Cpt1a* in the fat body. (**G**) Western blotting analysis of the IGF-2-like levels in the fat body of dsRNA injection. (**H**) Co-IP detected the interaction of IDE and CPT1A. Input: The levels of IDE and CPT1A in the fat body detected by an antibody against IDE or CPT1A. ACTB was a loading control. Co-IP: Anti-IDE antibody co-immunoprecipitated IDE. Nonspecific rabbit IgG was a negative control. Ten percent gel in SDS-PAGE. The data were analyzed by Student’s *t* test or ANOVA. The bars represent the means ± SD for three independent biological experiments.

Succinylation can regulate the function of IDE by regulating the subcellular localization, but the upstream regulatory mechanism of succinylation is unclear. To explore the mechanism of succinylation of IDE, we searched for enzymes related to succinylation modification. We interfered with the succinylation-related enzyme in the cells (fig. S6) and found that knocking down *Cpt1a* significantly reduced the succinylation level of IDE (Fig. 9A). Then, we knocked down *Cpt1a* via injection of *dsCpt1a* into sixth-instar 6-hour larval hemocoel. The fat-body tissue was detected after the first double-stranded RNA (dsRNA) injection at 96 hours. *Cpt1a* was confirmed as being knocked down significantly in the fat body (Fig. 9B). When *Cpt1a* was knocked down, the succinylation level of the IDE was reduced (Fig. 9C). Immunohistochemistry showed that the IDE of *dsGfp* injection group was mainly located in the nucleus and that of the *dsCpt1a* injection group was mainly located in the cytoplasm (Fig. 9D). Followed by Co-IP, the injection of *dsCpt1a* resulted in a significant decrease in IDE co-immunoprecipitated USP1, compared with injection of *dsGfp* (Fig. 9E). The expression of *Hr3* and *Br-Z7* was decreased after knockdown of *Cpt1a* in the fat body (Fig. 9F). Knockdown of *Cpt1a* resulted in significant reductions in the IGF-2-like levels (Fig. 9G). The protein interactions between IDE and CPT1A were examined by Co-IP. CPT1A was co-immunoprecipitated with IDE, compared with the negative IgG control (Fig. 9H). The specificity of CPT1A antibody was shown in fig. S7. These results suggested that CPT1A is involved in succinylation of IDE.

To further explore the regulatory effect of 20E on *Cpt1a*, 20E was injected into the larval hemocoel. In the fat body, *Cpt1a* expression was increased in a 20E concentration- and time-dependent manner (fig. S8, A and B), indicating that 20E promotes *Cpt1a* expression. On the basis of the JASPAR database, we found that the promoter region of CPT1A has two binding sites for KLF15(KLF15BS) 5′-CACCC-3′ (*4*), which is a transcription factor abundant in metamorphosis (fig. S8C). The expression of *Cpt1a* was decreased after knockdown of *Klf15* in the fat body (fig. S8D). Subsequently, we constructed the reporter plasmid *pCpt1a*-LUC-GFP-His and co-overexpressed it with KLF15-RFP-His in the cells (fig. S8E). Reporter assay showed that overexpression of KLF15-RFP-His under 20E treatment significantly up-regulated the expression of reporter genes (LUC-GFP-His), compared with overexpression RFP-His tag control (fig. S8F). The analysis of luciferase activity showed that overexpression of KLF15 under 20E treatment significantly enhanced the activity of firefly luciferase, with renilla luciferase as internal control (fig. S8G). A ChIP assay showed that both KLF15BS1 and KLF15BS2 of the promoter of *Cpt1a* can bind KLF15-RFP-His, and KLF15-RFP-His binds more KLF15BS motif under 20E treatment than DMSO control. RFP-His was used as a tag control, and, under DMSO or 20E treatment, RFP-His did not bind the KLF15BS motif (fig. S8H). The data confirmed that 20E up-regulates *Cpt1a* expression through KLF15.

## DISCUSSION

In insect metamorphosis development, the hydrolysis of larval tissue and the regeneration of adult tissue are carried out simultaneously. This process is regulated by 20E and ILPs, but the role of succinylation modification in this process is unknown. Here, LC-MS/MS identified 192 differentially succinylated proteins during insect metamorphosis. They were mainly enriched in glutathione metabolism, nutrient metabolism, and other metabolic pathways. Metalloproteinase IDE has attracted our attention as the intersection of succinylated differentially modified proteins associated with both ILPs and 20E. IDE can participate in different physiological processes by interacting with different targets, but the mechanism of target selection is not clear. This study provides evidence that non-succinylated IDE bind to IGF-2-like and degrade it during the feeding stage. 20E induces succinylation and nuclear localization of IDE-K179 during metamorphosis, leading to its interaction with the EcR-USP1 complex, enhancing 20E signaling pathway, and releasing of IGF-2-like to promote adult tissue proliferation and then promoting the metamorphosis of insects.

From prokaryotes to eukaryotes, lysine succinylome profiling analyses were performed in different species, such as 990 succinylated proteins were identified in *Escherichia coli*, 474 succinylated proteins were identified in yeast, 750 succinylated proteins were identified in mouse liver (*40*), and 164 succinylated proteins were identified in zebrafish (*41*). In insects, 373 succinylated proteins were identified in the mitochondria of the midgut of the fifth-instar silkworm, *B. mori* (*42*), but there is no report on succinylated modification proteomics related to insect metamorphosis. Using 6F and 6M cotton bollworm as samples, 523 succinylated proteins were identified, and the succinylated proteins with more than 1.3 times difference between the two stages were compared. A total of 181 proteins were down-regulated at the 6M stage, and 16 proteins were up-regulated at 6M stage. These differentially succinylated proteins may be involved in the metamorphosis of *H. armigera*.

Differential succinylated proteins are mainly located in the cytoplasm, which is consistent with the study in yeast, but, in mouse liver cells, succinylated proteins are mainly located in mitochondria (*40*), which may be related to the content of mitochondria in hepatocytes.

Eukaryotic cellular lysine succinylation is enriched on metabolic proteins and regulates nutrient metabolism and other processes (*43*, *44*). The role of succinylation in *H. armigera* corroborates the findings of a great deal of the previous work in other organisms. Among them, enzymes involved in glycolysis include fructose-bisphosphate aldolase, triose-phosphate isomerase, glyceraldehyde-3-phosphate dehydrogenase, phosphoglycerate mutase, and phosphopyruvate hydratase (*45*). Enzymes involved in TCA include citrate synthase, adenosine 5′-triphosphate citrate synthase, aconitate hydratase, isocitrate dehydrogenase, succinate–coenzyme A (CoA) ligase, fumarate hydratase, and malate dehydrogenase. Enzymes involved in fatty acid metabolism include 3-hydroxyacyl-CoA dehydrogenase, acyl-CoA dehydrogenase, and acetyl-CoA Acetyltransferase (*46*). Nutrient metabolism provides succinyl groups for succinylation, which, in turn, regulates the activity of enzymes in the nutritional metabolic pathway through covalent modification, which may also be a unique mechanism for precise regulation of metabolism.

KEGG enrichment analysis showed that differentially succinylated proteins were also enriched in cytochrome P450 involved in drug and xenobiotics metabolism, glutathione metabolism, and glyoxylate and dicarboxylate metabolism. The main differentially modified proteins involved in these metabolic pathways are cytochrome P450, glutathione transferase, and catalase, which are closely related to xenobiotics degradation and reactive oxygen species detoxification mechanisms (*47*, *48*). These functions of succinylated proteins were also reported in silkworm midgut (*42*) and rice that are resistant to *Magnaporthe oryzae* infection (*49*).

In addition to the metabolic pathways, this study focuses on differentially modified proteins associated with hormone signaling pathways that regulate insect metamorphosis. In the hormone-related differentially modified proteins mentioned in Fig. 2D, some have been identified with succinylation modifications. For example, lysine succinylation proteome in mouse cells found that hydroxyacyl-CoA dehydrogenase was highly succinylated with 32 sites and identified a number of highly connected subnetworks among Lys-succinylated proteins, including glutathione *S*-transferase (*50*). No succinylation modification was reported in other hormone-related proteins.

The most prominent of these differentially succinylated proteins is IDE, which is associated with both ILPs and 20E. IDE plays a role in the catabolism of bioactive peptides, especially insulin (*51*). Some studies have shown that knocking down IDE in *Drosophila* can increase the weight and fecundity of adults, decrease the level of glucose in hemolymph, and decrease the survival rate (*52*), which is similar to the phenotype caused by the imbalance of ILP signal in peripheral tissue (*53*, *54*), which indicates that IDE affects insect development by regulating ILPs levels. In addition, IDE can combine with AR or GR and enhance its binding ability with DNA (*18*). 20E belongs to steroid hormones as well as androgen and glucocorticoid, suggesting the possibility of IDE binding to EcRs. Considering that IDE is closely related to two hormones that regulate the development of insect metamorphosis, ILPs and 20E, we chose it as the starting point for the detailed mechanism of succinylation-regulating metamorphosis.

IDE is usually thought to be localized in the cytoplasm. Still, studies in rats have found that IDE can also be localized in the nucleus, and the nuclear component of IDE has no activity to degrade insulin, possibly due to other proteins competing with insulin to bind IDE (*18*). Moreover, inhibition of IDE activity is necessary to accumulate insulin in the nucleus (*55*). However, the mechanism and function of IDE localization in the nucleus are unknown.

PTM is an important way to regulate protein activity and subcellular localization rapidly. Succinylation modification has attracted more and more attention in recent years because it can change the lysine site’s charge from +1 to −1 under the physiological state, which changes the protein properties (*56*). Studies in colorectal cancer cell HCT116 have shown that succinylation modifications can alter the subcellular localization of the protein, the lysine mutation at position 433 of pyruvate kinase M2 to glutamate, mimic succinylation, and promote its localization to mitochondria (*10*). In in vivo experiments with the SV40 T antigen nuclear localization signal (NLS), it has been observed that sequences on both sides of NLS can affect the efficiency and rate of nucleation transport. Further studies have found that this effect is phosphorylation mediated; phosphorylation results in NLS exposure to accelerate nuclear entry (*57*).

Our results showed that succinylated IDE after 20E treatment can enter the nucleus from the cytoplasm, but the IDE-K179R mutant that mimics the non-succinylated state cannot enter the nucleus even in the presence of 20E, suggesting that succinylated modifications are important regulatory factors in the translocation of IDE from the cytoplasm into the nucleus. The results of bioinformatics analysis showed a segment of NLS with a high score at the amino acid positions 285 to 288 of IDE. It is speculated that succinylation of K179 may lead to a conformational transformation of the protein, exposing the NLS region and leading to its translocation from the cytoplasm into the nucleus.

IDE can interact with two steroid hormone receptors, AR and GR, and enhance their binding to DNA, but IDE itself does not directly bind to DNA (*18*). The downstream effects of IDE and steroid hormone receptor interactions are not well understood. Here, overexpressed mutants in cell lines and mutants introduced by gene editing systems in vivo demonstrated that succinylated IDE binds to steroid hormone 20E receptor complex EcR-USP1 to promote downstream gene expression. Non-succinylated IDE interacts with IGF-2-like, leading to its degradation. When the metamorphosis process is initiated, the succinylated IDE no longer binds to IGF-2-like. IGF-2-like was released to promote the proliferation of adult tissue. This study further elucidated the selection of IDE targets, and the molecular mechanism of IDE promotes metamorphosis. Using the HDOCK server (http://hdock.phys.hust.edu.cn/) (*58*), we assessed the potential of protein interaction by conducting molecular docking of the IDE-EcR complex and IDE–K179R–IGF-2-like complex. Physical binding interaction between IDE and EcR and between IDE-K179R and IGF-2-like was observed (fig. S9).

In addition to IDE, previous research showed that forkhead box class O (FOXO), a negative regulator of IIS/target of rapamycin, directly interacts with USP to mediate ecdysone biosynthesis (*59*). The synergistic regulation of 20E and IIS on insect metamorphosis may require the participation of more factors. The identification of these factors and the exploration of their relationships need further exploration.

Succinylation is the process by which a succinyl group donor covalently binds a succinyl group to a lysine residue of a protein by enzymatic or nonenzymatic means (*37*, *40*). The results of an in vitro experiment showed that, when different concentrations of Succinyl-Coenzyme A (Suc-CoA) were incubated with bovine serum albumin (BSA) and ovalbumin, the succinylation level increased with the concentration of Suc-CoA (*40*). It has also been found that, when α-Ketoglutarate Dehydrogenase Complex (KGDHC) is inhibited, the level of succinylation in mitochondria is proportional to the concentration of Suc-CoA. These results suggest that succinylation can occur through a nonenzymatic mechanism (*50*). This is due to the dicarboxylic acyl structure in Suc-CoA, which can form free CoA-SH and highly active acid cyclic anhydride, which reacts violently with the free ε-amino group of basic lysine (*60*). However, too much random succinylation in cells can lead to cell damage and metabolic disorders, so it is necessary to regulate succinylation through enzymes. In this study, the degree of succinylation of most proteins was higher in the feeding stage, which may be related to the abundance of metabolic intermediates in the feeding stage when larva consumed a large amount of food. At this point, succinylation can be performed in a nonenzymatic manner.

Because there were more cases of reduced succinylation in the experimental group, previous studies focused on the effect of reduced succinylation on protein function. However, there are also examples of elevated succinylation, such as the succinylation was found to increase with fasting in mice (*61*). The larvae stop feeding during metamorphosis, similar to the mouse’s fasting state, and the succinylation is likely to be catalyzed by enzymes. Several enzymes have been reported to be involved in the succinylation process, such as 3-oxoacid coenzyme A transferase 1, lysine acetyltransferase 2A, HAT1, and CPT1A (*62*, *63*). CPT1A is an enzyme responsible for transporting long-chain fatty acids into mitochondria during the oxidation of fatty acids (*13*, *64*, *65*). In 293T cells, CPT1A is also found to have lysine succinyltransferase activity (*13*). Various transcription factors have been shown to bind to the promoter of *Cpt1a*, such as AHR, SP1, and KLF15 (*66*, *67*). The molecular mechanism of steroid hormones regulating IDE succinylation is still unclear. It is speculated that the enzyme-transferring succinyl group is involved in this process. Interference experiments confirmed that CPT1A was engaged in the succinylation of IDE under 20E treatment. We also found KLF binding sites in the promoter region of *Cpt1a*. In *H. armigera*, 20E promotes autophagy, apoptosis, and gluconeogenesis by up-regulating KLF15 expression (*4*). Our study showed that, under the induction of 20E, KLF15 directly binds to the KLF binding site in the promoter region of *Cpt1a*, promoting the expression of *Cpt1a*. This study improved the molecular mechanism of IDE succinylation induced by 20E.

Last, succinylation modifies IDE lysine at site 179, which is evolutionarily conserved, providing potential for drug development targets. IDE exists in almost all eukaryotes and bacteria and has a highly conserved primary structure (*68*), which was originally identified as an IDE (*15*) and was involved in the efficient clearance of insulin to respond to the changes in blood sugar (*69*). In addition to insulin, IDE can also degrade other peptide hormones, such as amylin (*70*) and glucagon (*16*), indicating that IDE plays a complex role in the regulation of glucose metabolism. IDE dysfunction may lead to age-related glucose tolerance and type 2 diabetes mellitus (*70*). The accumulation of amyloid-β in the brain can lead to Alzheimer’s disease (*71*). IDE is also a major endogenous amyloid-β–degrading enzyme, which mediates the degradation and clearance of amyloid-β protein in the brain, thus reducing the toxicity of amyloid-β protein (*72*). IDE exists mainly in the cytoplasm, and it can also be located in many subcellular chambers, such as peroxisome, endosome, mitochondria, rough endoplasmic reticulum, and plasma membrane, and secreted to extracellular cells (*20*, *21*). Because of its close relationship with type 2 diabetes and Alzheimer’s disease, IDE has attracted much attention as a target for new drug development, but the mechanism of its binding with different targets has not been clear. Here, we found that succinylation can change the subcellular localization of IDE to bind to different target molecules. We identified the succinylation modification of the IDE at lysine-179. By sequence alignment, we found that lysine-179 in *H. armigera* was the same site as lysine-192 in *H. sapiens*, lysine-192 in *Mus musculus*, lysine-165 in *Drosophila melanogaster*, and lysine-180 in *B. mori*, indicating that succinylation at this site may be evolutionally-conserved (fig. S10). This evolutionary conservatism makes the results of this study provide a reference for the drug development of diabetes and Alzheimer’s disease targeting IDE.

**Fig. 10.**
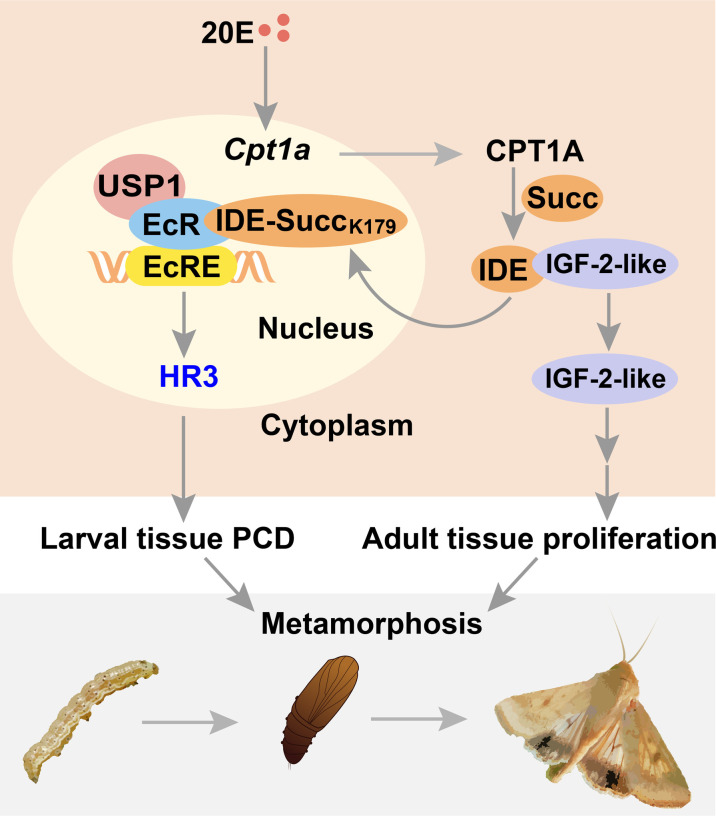
A diagram illustrating succinylation of IDE hubs destruction and reconstruction to regulate metamorphosis. Non-succinylated IDE localizes in the cytoplasm and binds to IGF-2-like. When the metamorphosis is initiated, 20E up-regulates *Cpt1a* expression, thus increasing the level of IDE succinylation on K179. Succinylated IDE translocates from cytoplasm to nucleus, combined with EcR complex to promote PCD of larval tissues; meanwhile, IGF-2-like is released to promote adult tissue proliferation. Therefore, succinylation alters subcellular localization of IDE, so that it can bind to different target proteins and act as a hub mediating the coordination of 20E and ILPs in insect metamorphosis.

In conclusion, succinylome profiling analysis identified IDE as a hub in that 20E and ILPs coordinated larval tissue destruction and adult tissue reconstruction simultaneously. The results show that non-succinylated IDE localizes in the cytoplasm, binds to IGF-2-like, and degrades it during feeding stage. When the metamorphosis is initiated, 20E up-regulates *Cpt1a* through transcription factor KLF15 and induces the succinylation of IDE-K179. Succinylated IDE translocated from cytoplasm to nucleus, combined with EcR-USP1 to enhance 20E signaling pathway and released IGF-2-like to promote adult tissue proliferation and then to promote the metamorphosis of insect. The results of this study provide evidence that hormones regulate IDE subcellular localization at the PTM level to act on specific targets and provide reference for drug development targeting IDE (Fig. 10).

## MATERIALS AND METHODS

### Experimental animals

Cotton bollworms (*H. armigera*) were reared on an artificial diet in our laboratory at 27° ± 1°C with 60 to 70% humidity and under a 14-hour light/10-hour dark photoperiod. The larvae were fed on an artificial diet containing soy flour, wheat germ, multivitamins, sucrose, and inorganic salts (*73*).

### Experimental cells

The *H. armigera* epidermal cell line (HaEpi) was established and has been well characterized previously (*74*). HaEpi cells were maintained in Grace’s medium (Gibco, California, USA) supplemented with 10% fetal bovine serum (Biological Industries, Cromwell, CT, USA) at 27°C.

### Bioinformatic analysis

The phylogenetic tree was constructed using MEGA 7.0 software with the neighbor-joining method. The protein structural domains and motifs were predicted using SMART (http://smart.embl-heidelberg.de/). The molecular characterization of proteins was performed using the ExPASy ProtParam tool (https://web.expasy.org/protparam/). The DNAMAN software (Lynnon Biosoft, San Ramon, CA, USA) was used to generate the multiple sequence alignment. The binding motifs were predicted by the JASPAR transcription factor database (http://jaspar.genereg.net/).

### Succinylome profiling analysis

The life cycle of cotton worm consists of four stages: egg, larva, pupa, and adult. The larva stage was segmented into six instars by molts. After eating for more than 48 hours, sixth-instar larvae got into the metamorphic commitment stage at 72 hours and then pupation at about 144 hours. We compared the protein expression profile and succinylation level in the whole bodies of bleeding larvae between sixth-instar feeding (6F, 12 to 60 hours after ecdysis into sixth instar) and metamorphic commitment stages (6M, 72 to 120 hours after ecdysis into sixth instar).

After digestion by trypsin, the peptides were dissolved in Nonidet P-40/EDTA/Triton X-100/NaCl (NETN) buffer [100 mM NaCl, 1 mM EDTA, 50 mM tris-HCl, and 0.5% NP-40 (pH 8.0)], and the supernatant was transferred to prewashed anti–succinyl-lysine antibody coupling resin (PTM Bio, Hangzhou, China) at 4°C overnight with gentle shaking. Then, the resin was washed four times with NETN buffer and twice with deionized water. The resin-bound peptide was eluted with 0.1% trifluoroacetic acid, and the eluent was collected and vacuum dried. The rest of the detailed procedure of label-free quantitative proteomics, including protein extraction, trypsin digestion, LC-MS/MS, and bioinformatics analysis, has described in our previous work (*75*).

### Preparation of rabbit polyclonal antiserum against IDE

A fragment of *Ide* [nucleotide sequence 2445 to 3006 base pairs (bp), amino acids 815 to 1002] was amplified using primers Ide-ExpF and Ide-ExpR (table S3). The polymerase chain reaction (PCR) product was inserted into the pET-30a expression vector. The recombinant plasmid was expressed in *E. coli* strain Rosetta (DE3) with 0.1 mM isopropyl β-d-thiogalactoside induction. Recombinant IDE protein was purified using a Ni^2+^–nitrilotriacetic acid affinity column (GE Healthcare, Chicago, USA). Rabbits were immunized by the purified antigen to obtain rabbit polyclonal antibodies (anti-IDE antibodies). The specificity of the antiserum was determined using Western blotting.

### Western blotting

Total proteins were extracted from cells or larval tissues using phosphate-buffered saline (PBS) or tris-HCl buffer (pH 7.5, 40 mM) with 1 mM phenylmethylsulfonyl fluoride. The samples were centrifuged at 10,000*g* at 4°C for 15 min, the supernatant was retained, the protein concentration was determined using the bicinchoninic acid (BCA) method, and the supernatant and protein loading buffer were incubated in a 100°C water bath for 15 min. Total proteins (20 μg) were separated by SDS–polyacrylamide gel electrophoresis (SDS-PAGE) and then transferred onto a nitrocellulose membrane. The membrane was incubated in blocking solution prepared with TBST [0.02% Tween in tris-buffered saline (TBS); 10 mM tris-HCl and 150 mM NaCl (pH 7.5)] for 1 hour at room temperature and then incubated at 4°C overnight with the primary antibodies in blocking buffer. The nitrocellulose membrane was washed two times for 10 min each with TBST and once for 10 min with TBS, followed by incubation for 2 hours at room temperature with the horseradish peroxidase–labeled goat anti-rabbit/mouse immunoglobulin G (IgG)/IgG (light-chain) secondary antibodies (ZSGB-BIO, Beijing, China) diluted 1:7000 in blocking buffer. The membrane was washed twice with TBST for 10 min each time, followed by TBS for 5 min once. The membrane was immersed in High-sig ECL Western blotting substrate (Tanon Science & Technology, Shanghai, China), and the immunoreactive protein bands were visualized using the electro-chemiluminescence (ECL) luminescence method. The protein bands were detected using a 5200 chemiluminescence imaging system (Tanon Science & Technology, Shanghai, China). The densities of the bands were analyzed using ImageJ software (National Institutes of Health, Bethesda, MN, USA).

### Whole-mount immunohistochemistry

The fat body was rinsed in PBS and then fixed in 4% paraformaldehyde for 12 hours at 4°C. The samples were blocked with 5% BSA (PBS with 0.3% Triton X-100) for 3 hours and then incubated with a primary antibody against IDE at 4°C for 24 hours. Alexa Fluor 488–conjugated goat antirabbit IgG secondary antibodies (Abbkine, California, USA) were used and incubated at 37°C for 1 hour. The fat-body samples were rinsed with PBS. The dye of 4′,6-diamidino-2-phenylindole (DAPI; 1 μg/ml in PBS, Sangon, Shanghai, China) was used for nuclear staining in the dark for 20 min and then washed with PBS three times. The image was showed using an Olympus BX51 fluorescence microscope (Olympus Optical Co., Tokyo, Japan).

### Overexpression of proteins in HaEpi cells

The open reading frames of the target sequence were amplified by PCR using the corresponding primers (table S3) and then inserted into pIEx-4-GFP-His vector to overexpress the target protein with C-terminal GFP and histidine tags. HaEpi cells were grown in six-well plates to 80% confluence, the medium was replaced with fresh medium, 5 μg of plasmid and 8 μl of Quick Shuttle–enhanced transfection reagents were transfected into the HaEpi cells for 48 hours, and then subsequent experiments were carried out.

### Immunocytochemistry

HaEpi cells were grown on a density of 2 × 10^5^ in a cell culture plate. After the plasmid was overexpressed for 48 hours, the cells were incubated in 2 μM 20E for 1 hour. HaEpi cells were washed three times with DPBS [137 mM NaCl, 2.7 mM KCl, 1.5 mM KH_2_PO_4_, and 8 mM Na_2_HPO_4_ (pH 7.4)] and fixed with 4% paraformaldehyde for 10 min in the dark at room temperature. The fixed cells were washed six times for 3 min each. Nuclei were stained with DAPI (Sangon, Shanghai, China; 1 μg/ml in PBS) in the dark at room temperature for 10 min and then washed with PBS three times. Fluorescence was detected using an Olympus BX51 fluorescence microscope (Olympus Optical Co., Tokyo, Japan).

### Co-immunoprecipitation

The total proteins were extracted accordingly using radioimmunoprecipitation assay (RIPA) buffer (Beyotime, Shanghai, China) with protease inhibitors, and, then, the supernatant was collected via centrifugation at 10,000*g* for 15 min at 4°C. The 50-μl supernatant was used for Western blotting analysis. Then, protein A resin was washed three times for 5 min each with lysis buffer. The supernatant was incubated with protein A resin to eliminate nonspecific binding and collected by centrifugation (1000*g* for 5 min). The supernatant and the antibodies were incubated for 4 hours with gentle shaking at 4°C. The protein A resin was then incubated with the protein-antibody complex with gentle shaking for 2 to 4 hours at 4°C. The resin was subsequently collected via centrifugation at 3000*g* for 5 min and washed three times with RIPA buffer. Last, the collected resin was treated with SDS-PAGE loading buffer and boiled for 10 min. The samples were subjected to SDS-PAGE for Western blotting and analysis using target antibodies to detect the target proteins, respectively.

### Luciferase reporter assay

The promoter sequences of *Cpt1a* were inserted into pIEx-4-Luciferase-GFP-His (LUC-GFP-His) to construct *pCpt1a*-LUC-GFP-His. The binding sites were predicted according to the JASPAR website (https://jaspar.genereg.net/). KLF15-RFP-His was cotransfected with *pCpt1a*-LUC-GFP-His. After 24 hours of cotransfection, cells were incubated with 20E (2 μM) or DMSO for 24 hours. Western blotting analyzed the expression of LUC-GFP-His. KLF15-RFP-His, pRL-TK, and *pCpt1a*-LUC-GFP-His were cotransfected for 24 hours and then were incubated with 20E or DMSO for another 24 hours. Luciferase activity was measured by a Dualucif Firefly and Renilla Assay Kit (UElandy, Suzhou, China) on the basis of the instructions.

### Chromatin immunoprecipitation

The plasmid was transfected into HaEpi cells for 72 hours, which were then treated with 2 μM 20E for 6 hours, and DMSO was used as the control. The samples were treated with the ChIP assay kit (Beyotime, Shanghai, China) according to the instructions. Anti-His antibody was used to detect the target protein, and IgG was used as a negative control. The DNA was purified using phenol/chloroform extraction and analyzed by quantitative reverse transcription (qRT)–PCR with the corresponding primers (table S3).

### Real-time qRT-PCR

Total RNA was extracted from the cell or larvae using the TRIzol reagent (TransGen Biotech, Beijing, China), then the RNA concentration was detected, and 2 μg of RNA transcribed to first-strand cDNA was quantified using a 5× All-In-One RT Master Mix kit (Applied Biological Materials, Richmond, Canada) according to the manufacturer’s instructions. qRT-PCR was performed with the qTOWER3/G system (Analytik Jena AG, Jena, Germany), containing 5 μl of TransStart Tip Green qPCR Supermix (Aidlab, Beijing, China), 1 μl of cDNA, and 2 μl each of the forward and reverse primers (1 μM), as listed in table S3. The *Actb* (β*-actin*) was used as the internal control. Data were analyzed by the formula: ΔΔCT = ΔCT_treated sample_ − ΔCT_control_, ΔCT = CT_gene_ − CT_Actb_ (*76*).

### dsRNA synthesis

The effects of RNA interference (RNAi) have been validated in a variety of moths (*77*). In the worm, the long dsRNA is broken down into smaller fragments in vivo; it then specifically inhibits the expression of target genes (*78*, *79*). DNA template was amplified using RNAi primers (table S3). The 50 μl dsRNA transcription system contains 2 μg of DNA template, 20 μl of 5× transcription buffer, 3 μl of T7RNA polymerase (20 U/μl), 2.4 μl of A/U/C/GTP (10 mM) each, 3 μl of ribonuclease (RNase) inhibitor (40 U/μl, Thermo Fisher Scientific, Waltham, USA), and RNase-free water. The sample was incubated at 37°C for 4 to 6 hours, and 10 μl of RNase-free deoxyribonuclease I (DNase I; 1 U/μl; Thermo Fisher Scientific), 10 μl of DNase I buffer, and 30 μl of RNase-free water were added to the solution, which was incubated at 37°C for 1 hour. The dsRNA was purified with phenol/chloroform and precipitated with ethanol; the precipitate was resuspended in 50 μl of RNase-free water. The purity and integrity of the dsRNA were confirmed using agarose gel electrophoresis. The concentration of dsRNA was detected using a MicroSpectrophotometer (GeneQuant; Amersham Biosciences, Little Chalfont, UK).

### RNAi in HaEpi cells and larvae

The target of dsRNA (2 μg) was transfected into HaEpi cells using the Quick Shuttle–enhanced transfection reagent (Biodragon, Beijing, China) in 1.5 ml of Grace’s medium, according to the manufacturer’s instructions. *dsGfp* was used as the control group. After 48 hours of dsRNA treatment, 20E or an equivalent amount of DMSO was added to the cell culture medium at the concentration and time required for the experiment. The sample was then extracted for analysis. The larvae were injected with dsRNA (1 μg per larva) into the hemocoel, three times at 24 hours apart. The control group was treated with the same amount of *dsGfp*. The interference efficiency, the expression of the target gene, and the morphology of the fat body were determined after the last injection of dsRNA.

### Editing *Ide* using the CRISPR-Cas9 system

gRNAs were designed using the CRISPRscan tool (www.crisprscan.org/?page=sequence) (*80*). The gRNA consisted of an ~20-nucleotide region in complementary reverse to one strand of the target DNA next to an NGG motif (PAM) and the T7 polymerase binding site. The single-guide RNA (sgRNA) primer and the universal primer were used to obtain amplification products. The template transcription was carried out with a T7 Transcription Kit (Thermo Fisher Scientific, Waltham, USA) according to the manufacturer’s instructions. The donor plasmid was designed to introduce three mutant sites and homologous arm (~1500 bp, left and right ends of the target site). The donor plasmid serves as a template of homology-directed repair for repairing the Cas9-created double-strand break. Freshly laid eggs (within 2 hours) were collected and affixed onto microscope slides (*81*, *82*). A mixture of Cas9 protein (200 ng/μl; GenScript, New Jersey, USA), donor DNA (200 ng/μl), and gRNA (400 ng/μl) was injected into the eggs (per egg, 2 nl was injected) using a picoliter microinjector (Warner Instruments, Holliston, USA) (*83*). The injected eggs were incubated at 26° ± 1°C with 60 ± 10% relative humidity. For identification of the mutations of *H. armigera*, primers Ide-test-F and Ide-test-R (table S3) were used to amplify a ~300-bp DNA fragment flanking the target site of sgRNA. PCR amplification of the targeted genomic region was carried out by fresh epidermis samples of molted larvae. The PCR products were sequenced, and the PCR fragments from the mutant animals were ligated into a pMD19-T vector (TaKaRa, Osaka, Japan) for sequencing. The mutated sites were identified by comparison with the wild-type sequence.

### H&E staining

The fat-body tissue was rinsed with PBS and fixed in 4% paraformaldehyde at 4°C for 12 hours. The fixed tissues were dehydrated gradually with different buffers. The fat-body tissue was embedded in paraffin, and the paraffin was sliced using a paraffin-slicing machine. The sections were flattened and adhered to a glass slide in a 60°C water bath. The slides were dried at 60°C for 6 hours and subsequently dewaxed. The sample sections were rehydrated gradually and digested with proteinase K (20 μg/ml) at 37°C for 10 min. The sample sections were stained using an H&E Stain Kit (Solarbio, Beijing, China) following to the manufacturer’s instructions. The H&E staining images were observed using an Olympus BX51 fluorescence microscope (Olympus Optical Co., Tokyo, Japan).

### Nile red staining

The frozen slices were dried at room temperature. The slides were fixed in 4% paraformaldehyde solution at room temperature for 30 min. The sections were washed with PBS three times. The proteinase K solution (20 μg/ml, 50 μl) was added to each sample and incubated for 20 min at room temperature, and, then, sections were washed with PBS. The tissue was incubated with Nile red (MCE, New Jersey, USA) solution at 37°C for 1 hour in the dark. The dye solution was removed and washed three times in the dark with PBS. The DAPI (BBI LIFE SCIENCE, Shanghai, China) solution was incubated in the dark for 10 min. The dye solution was removed and washed three times in the dark with PBS. The slides were sealed and observed using an Olympus BX51 fluorescence microscope (Olympus Optical Co., Tokyo, Japan).

### Protein-protein docking

The protein three-dimensional structure was simulated using I-TASSER (https://zhanggroup.org/I-TASSER/), and an HDOCK server (http://hdock.phys.hust.edu.cn/) was applied to predict the molecular docking. HDOCK server predicts protein-protein interaction through a hybrid algorithm of template-based docking, and the top 10 docking results can be visualized on the result web page. The protein-protein complex with the highest negative docking scores was assessed for the docking poses. PyMOLv2.5 was used to visualize the protein-protein complex structure and to label structural features.

### The antibodies used in this study

Polyclonal anti-IDE antibody was prepared in our laboratory using recombinantly expressed protein in *E. coli*. The specificity of the antibody was detected by Western blotting. Polyclonal anti-succinylation antibody (RRID: AB_2687628) was obtained from Jingjie PTM BioLab Co. Ltd. (Jingjie PTM BIO, Hangzhou, China). Monoclonal anti-GFP antibody (RRID: AB_2770402), monoclonal anti-RFP antibody (RRID: AB_2770409), and polyclonal anti-ACTB antibody (RRID: AB_2768234) were obtained from ABclonal Technology (ABclonal, Wuhan, China). Polyclonal anti–IGF-2-like antibody was prepared in our laboratory according to the report (*39*). Polyclonal anti-CPT1A antibody (RRID: AB_2687628) was obtained from Proteintech Group Inc. (Proteintech, Rosemont, USA).

### Statistical analysis

All data were collected from at least three biological replicates. The density of Western blotting protein bands was analyzed using ImageJ software (National Institutes of Health, http://imagej.nih.gov/ij/download.html). The GraphPad 8 software (GraphPad Inc., La Jolla, CA, USA) was used to analyze data and to generate the figures. The two-group datasets were analyzed by Student’s *t* test (*P < 0.05 and **P < 0.01). The analysis of variance (ANOVA) was applied for analysis of multiple sets of data. The different letters show significant differences (*P* < 0.05). The bars indicate the means ± SD of three biological replicates.
