# Supplementary material for: Succinylation enables IDE to act as a hub of larval tissue destruction and adult tissue reconstruction during insect metamorphosis
Source: Sci Adv. 2025 Feb 5;11(6):eads0643. doi: 10.1126/sciadv.ads0643 (PMC11797550; doi:10.1126/sciadv.ads0643)
Supplement: Supplementary file 1 — Figs. S1 to S10 Tables S2 and S3 Legend for table S1 [file sciadv.ads0643_sm.pdf]

Science Advances
AAAS

Supplementary Materials for

Succinylation enables IDE to act as a hub of larval tissue destruction and adult tissue reconstruction during insect metamorphosis

Yan-Xue Li *et al.*

Corresponding author: Du-Juan Dong, dongdj@sdu.edu.cn

Sci. Adv. **11**, eads0643 (2025)
DOI: 10.1126/sciadv.ads0643

The PDF file include:

Figs. S1 to S10
Tables S2 and S3
Legend for table S1

Other Supplementary Material for this manuscript includes the following:

Table S1

Supplementary figures and legends

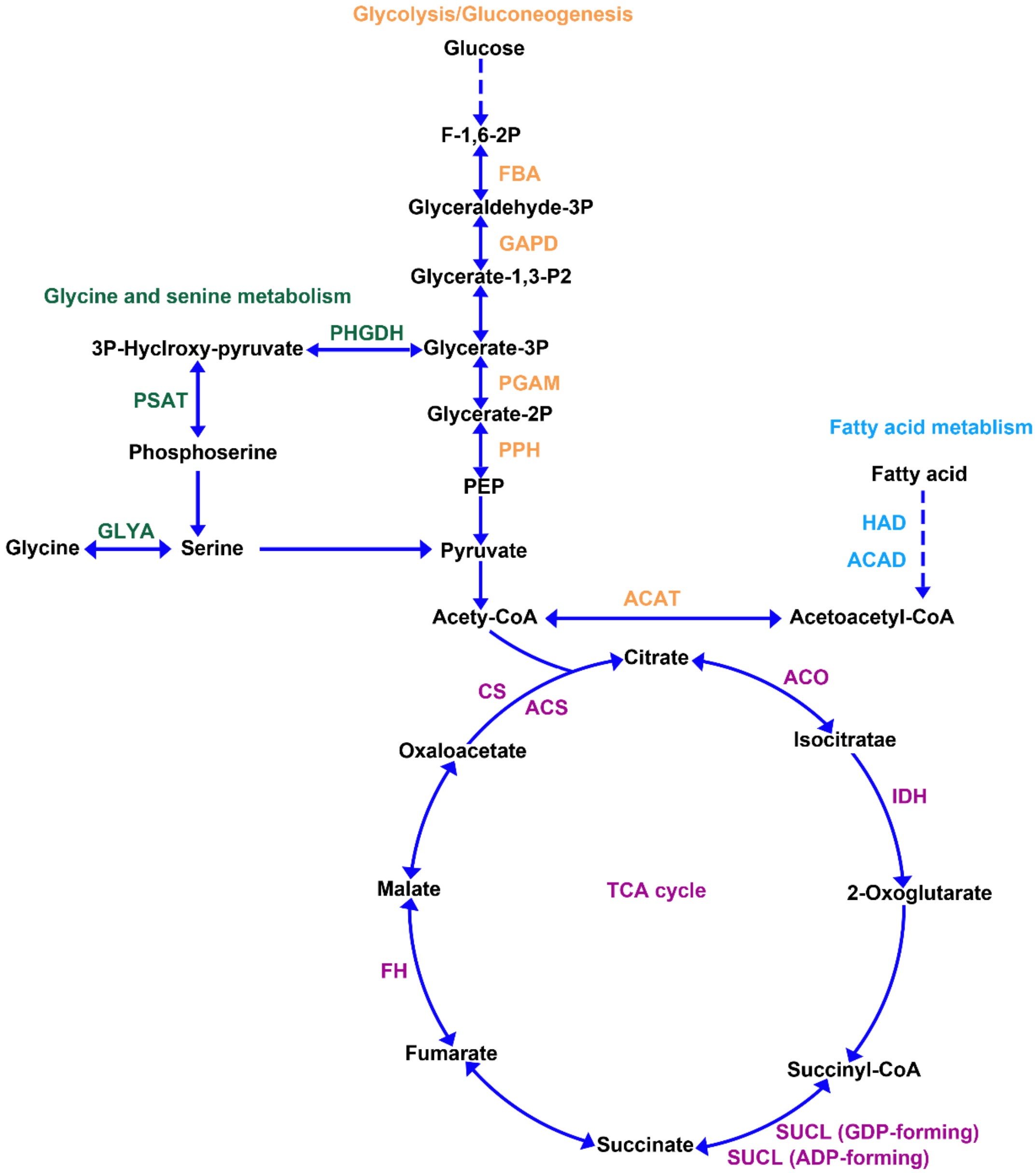

Fig S1. The diagram shows the succinylated proteins involved in metabolism. TCA cycle, tricarboxylic acid cycle; FBA, fructose-bisphosphate aldolase; GAPD, glyceraldehyd-3-phosphate dehydrogenase; PGAM, phosphoglycerate mutase; PPH, phosphopyruvate hydratase; ACAT, acetyl-CoA C-acetyltransferase; HAD, 3-hydroxyacyl-CoA dehydrogenase; ACAD, acyl-CoA dehydrogenase; CS, citrate synthase; ACS, ATP citrate synthase; ACO, aconitate hydratase; IDH, isocitrate dehydrogenase; SUCL, succinate-CoA ligase; FH, fumarate hydratase; PHGDH, phosphoglycerate dehydrogenase; PSAT, phosphoserine aminotransferase; GLYA, glycine.

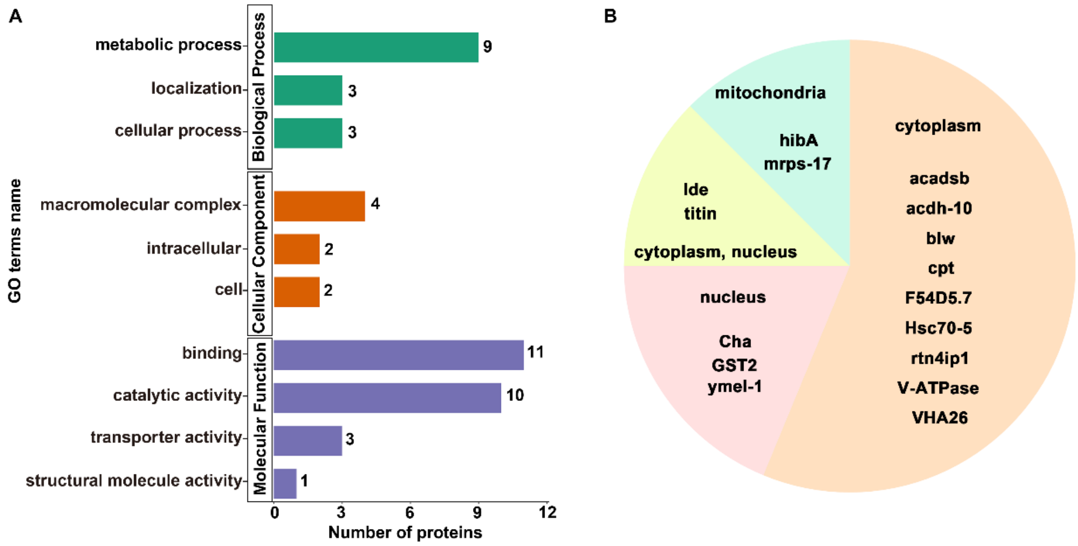

Fig S2. Function and localization analysis of proteins with increased succinylation during metamorphosis. A. Enrichment analyses of the up-regulated succinylated proteins in biological effect based on GO. **B**. Subcellular locations of up-regulated succinylated proteins.

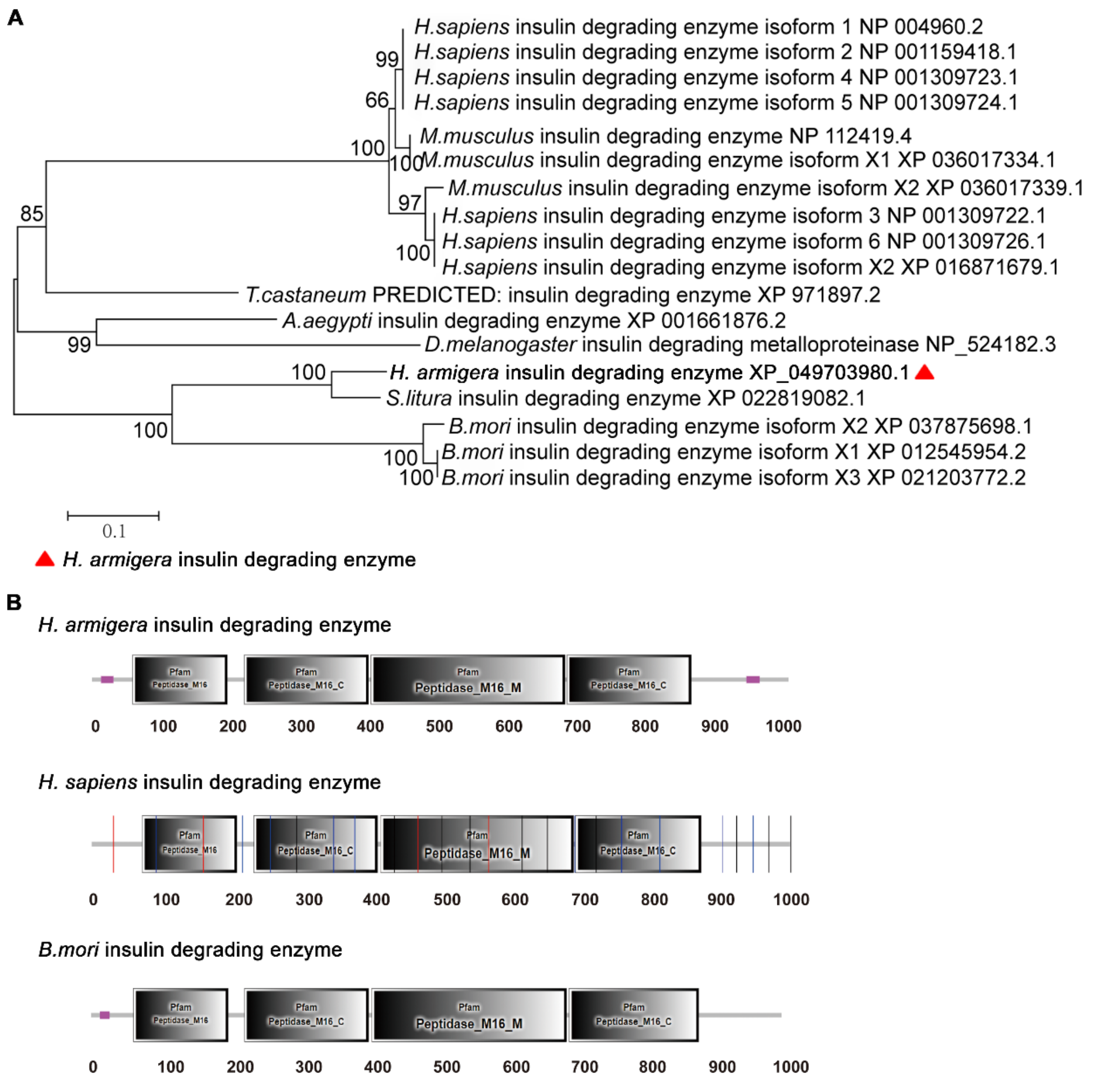

Fig S3. Phylogenetic tree and domain analysis of IDE. A. Phylogenetic tree analysis of IDE in multiple species. **B.** Domain prediction of IDE in *H.armigera*, *H.sapiens*, and *B.mori*. The black box represents, in turn, Peptidase_M16, Peptidase_M16_C, Peptidase_M16_M, and Peptidase_M16_C superfamily.

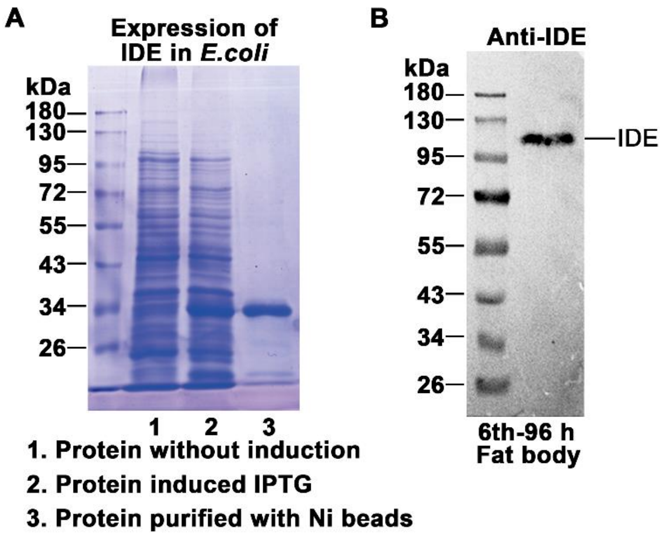

Fig S4. The polyclonal rabbit antibodies against *H. armigera* IDE. A. IDE recombinantly expressed protein in *E. coli*. **B.** The specificity of IDE polyclonal antibodies was analyzed by western blotting.

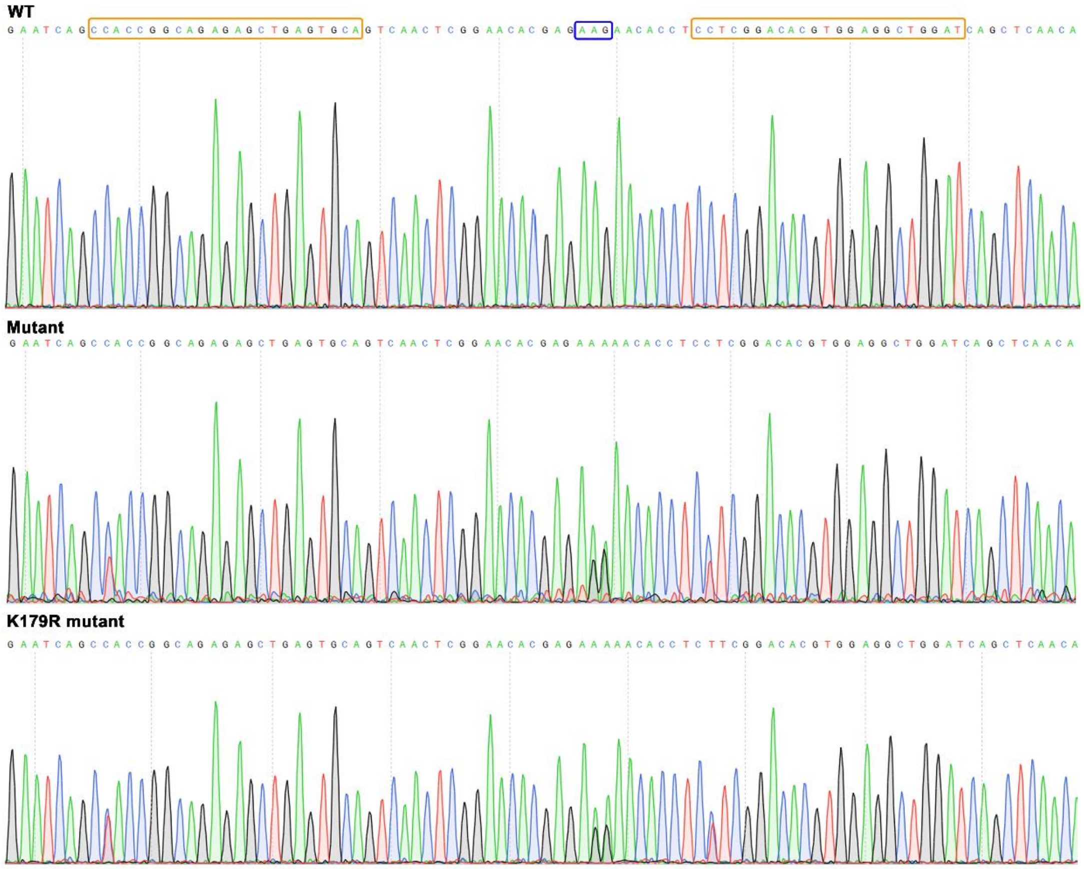

Fig S5. Representative chromatograms of the PCR products were used to determine genotype. The orange box represents the gRNA site and the blue box represents the K179 site.

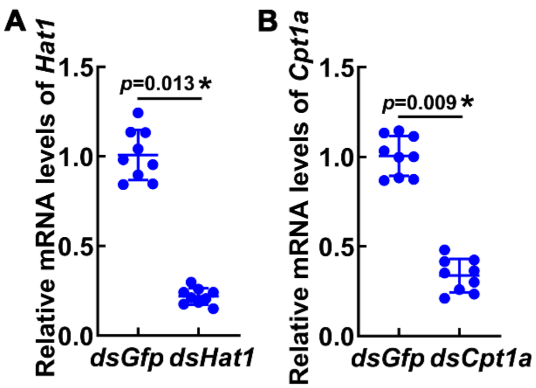

Fig S6. A. qRT-PCR analysis of the interference efficiency of *Hat1* and *Cpt1a*. A. The expression level of *Hat1* after knockdown *Hat1* in HaEpi cells. *dsGfp* as a control. **B.** The mRNA levels of *Cpt1a* were detected by qRT-PCR after knockdown in HaEpi cells.

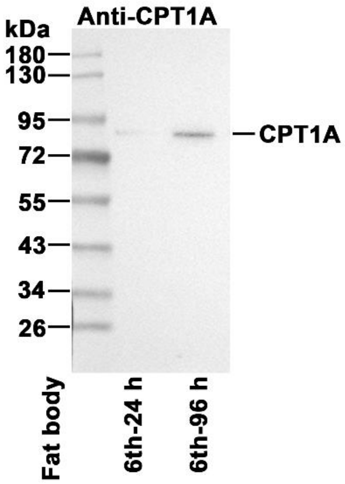

Fig S7. The specificity of CPT1A polyclonal antibodies. The specificity of CPT1A polyclonal antibodies was analyzed by western blotting. Samples were taken from fat body of 6th-24 h and 6th-96 h.

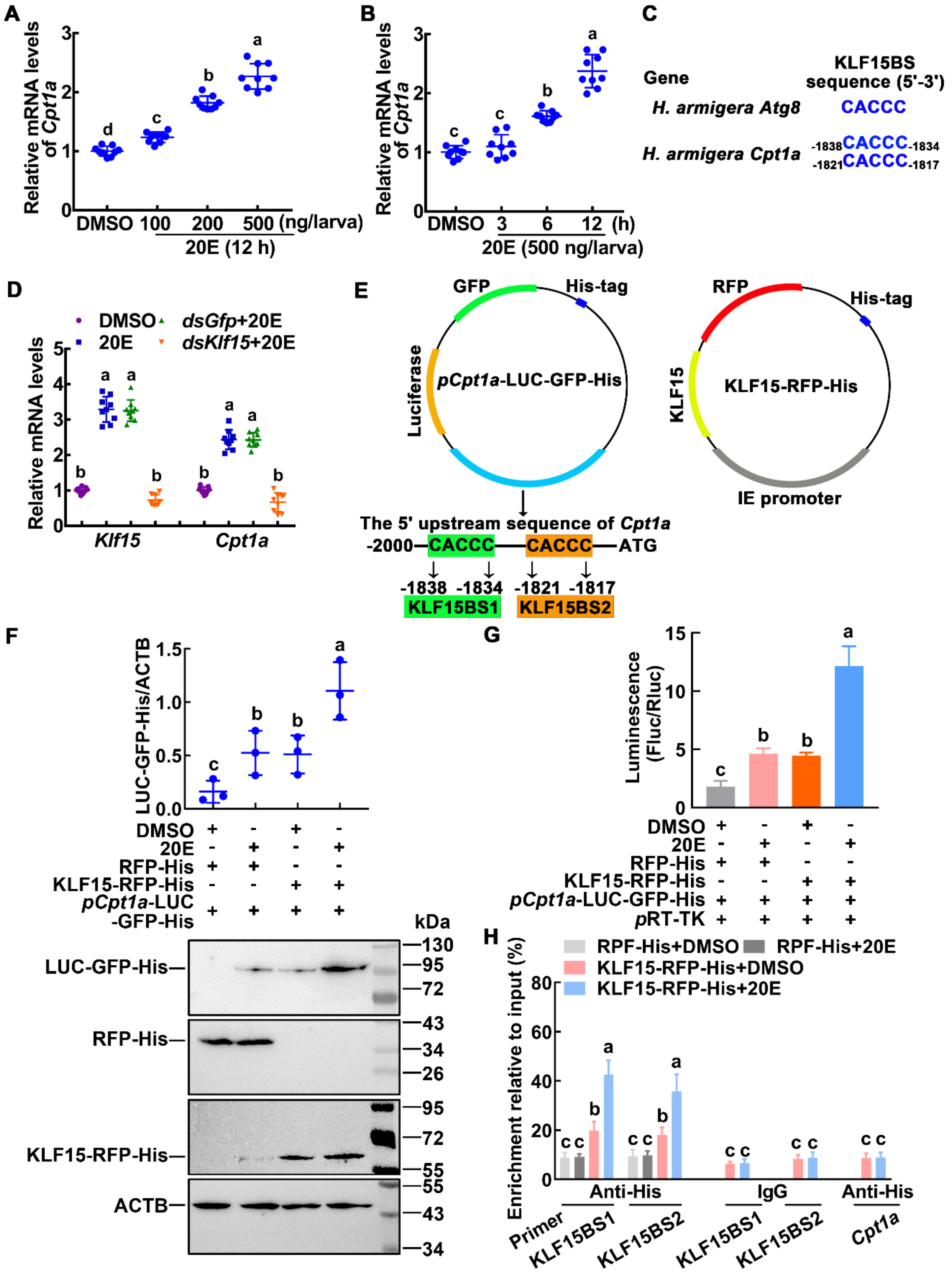

Fig S8. 20E up-regulated the expression of *Cpt1a* via KLF15. A. The expression of *Cpt1a* in the fat body under stimulation with different concentrations of 20E for 12 h. **B.** Time course of the *Cpt1a* expression in the fat body after 20E (500 ng/larva) induction. DMSO was used as the control. **C.** KLF15 binding site (KLF15BS) of *Atg8* and *Cpt1a* promoter region of *H. armigera*. **D.** The expression of *Cpt1a* after knockdown of *Klf15* in the fat body by *dsKlf15* (1 μg/larva) followed by

stimulation with 20E (500 ng/larva) for 12 h. **E.** The map of *Cpt1a* reported plasmid (*pCpt1a*-LUC-GFP-His) and KL15 overexpression plasmid (LKF15-RFP-His). **F.** Western blotting showed the expression of the *pCpt1a*-LUC-GFP-His reporter plasmid (LUC-GFP-His) under overexpression of KLF15-RFP-His or RFP-His with 20E (2 μM, 24 h) or DMSO treatment. **G.** The fluorescence of the firefly (Fluc) represents reporting activity, and the fluorescence of Renilla (Rluc) was used as an internal reference to eliminate the background. The ratio of Fluc to Rluc represents the relative luminescence intensity. **H.** ChIP assay showed that 20E promotes *Cpt1a* expression via KLF15 binding to KLF15 binding site (KLF15BS). The primers for KLF15BS are the sequences containing KLF15BS in the *Cpt1a* promoter region, respectively. Primer *Cpt1a* targeting the *Cpt1a* CDS was used as a control. The data was analysis by ANOVA. The bars represent the mean ± SD for three independent biological experiments.

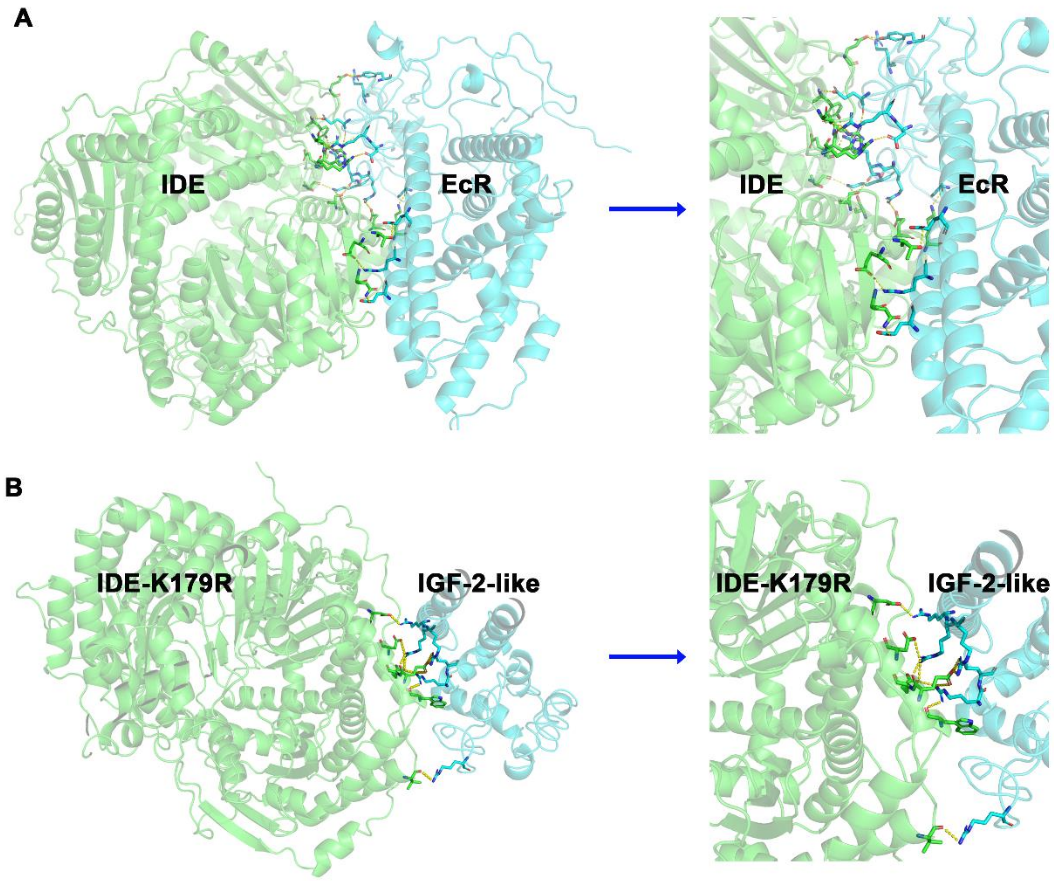

Fig S9. Graphical exhibition of modeling of the protein-protein complex. A. Modeling of the IDE-EcR complex. Green: IDE; Blue: EcR. The dotted lines (yellow) indicate the predicted hydrogen bonds between the amino acid residues. **B.** Modeling of the IDE-K179R and IGF-2-like docking. Green: IDE-K179R; Blue: IGF-2-like. The dotted lines (yellow) indicate the predicted hydrogen bonds between the amino acid residues.

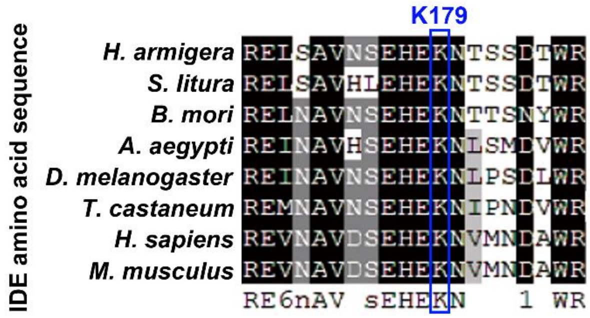

Fig S10. Conservative comparison of succinylation modification sites. The degree of black represents the degree of conserved, amino acid sequence from the following species: *H. armigera* IDE, *S. litura* IDE (XP_022819082.1), *B. mori* IDE (XP_012545954.2), *A. aegypti* IDE (XP_001661876.2), *D. melanogaster* IDE (NP_524184.3), *T. castaneum* IDE (XP_971897.2), *H. sapiens* IDE (NP_004960.2), *M. musculus* IDE (NP_112419.4).

Table S2. The list of different succinylated proteins involved in ILP or steroid hormone signaling pathways.

Steroid hormone pathway		Insulin-like peptide pathway	
**Name**	**Reference**	**Name**	**Reference**
Rho GDP-dissociation inhibitor (RhoGDI)	*(22)*	Peroxiredoxin (PRDX)	*(28)*
Heat shock 70 kDa protein (HSP70)	*(23)*	Triosephosphate isomerase (TPI)	*(29)*
Filamin-A (FLNA)	*(24)*	3-hydroxyacyl-CoA dehydrogenase (3-HADH)	*(30)*
Glutathione S-transferase (GST)	*(25)*	Succinyl-CoA:3-ketoacid coenzyme A transferase (SCOT)	*(31)*
Annexin (ANX)	*(26)*	Phosphoserine aminotransferase (PSAT)	*(32)*
Calponin (CALP)	*(27)*	Trypsin (TPS)	*(33)*
Insulin-degrading enzyme (IDE)	*(18)*	14-3-3 protein (14-3-3 protein)	*(34)*
		Elongation Factor 1 alpha (EF-1 alpha)	*(35)*
		Insulin-degrading enzyme (IDE)	*(36)*

Table S3. Oligonucleotide sequences of PCR primers.

Primer name	5′ ⟶ 3′ nucleotide sequence
**qRT-PCR**	
Ide-RTF	tatccagtatgtcgtcccaa
Ide-RTR	caatgttgtcgtatcgcttg
Hr3-RTF	tcaagcacctcaacagcagcccta
Hr3-RTR	gaactttgctgatgtcaccctccgc
Br-Z7-RTF	atggctgatcaattctgttta
Br-Z7-RTR	gttcggtgaagagaaattttc
Wnt-RTF	gtcacggtatgtcaggctcg
Wnt-RTR	ggctccaggtcagtattaggc
c-Myc-RTF	atgaagactggaggataatgga
c-Myc-RTR	tgttgtgggtgtagtggttg
Hat1-RTF	accgctactacgcctacccg
Hat1-RTR	tgattcgccatttcttctga
Cpt1a-RTF	atacagcatcgcccaaaag
Cpt1a-RTR	cgggagaaatccgacaact
Klf15-RTF	tgttttgtgaaggctctggat
Klf15-RTR	gatctaaatcactaagctgcc
β-actin-RTF	cctggtattgctgaccgtatgc
β-actin-RTR	ctgttggaaggtggagagggaa
**RNAi**	
Hat1-RNAiF	gcgtaatacgactcactatagggggatttgtgctcagttgct
Hat1-RNAiR	gcgtaatacgactcactataggcggcggtactcgtgttctag
Cpt1a-RNAiF	gcgtaatacgactcactataggcaggtcatcgcttgtttatt
Cpt1a-RNAiR	gcgtaatacgactcactatagggtatgttggtgggtctcagt
Klf15-RNAiF	gcgtaatacgactcactataggtctcctgcggaaagtcttata
Klf15-RNAiR	gcgtaatacgactcactataggcctctactgggcaacgaaaa
**Expression**	
Ide-Exp-F	cggggtacccaactgggctacatagtcttca
Ide-Exp-R	cgggatatcgtgcgggtataacggtctt
**Overexpression**	
Ide-Oexp-F1	gcatcgttaacacgtcaagagctcatgtcgtcccagttctctccc
Ide-Oexp-R1	catggtaccgtcgacctgcagaagtttacactgcgatccttttct
**Gene editing**	taatacgactcactatagtgcactcagctctctgccgggttttagagctagaa
gRNA1	taatacgactcactatagatccagcctccacgtgtccggttttagagctagaa
gRNA2	tgattatgaatgacacacccg
Ide-test-F	gacagacagacagacccacct
Ide-test-R	aaaccggcttcgagttctgt
Donor-F	ttcctgacatcaatcccttt
Donor-R	
**Luciferase**	
pCpt1a-LUC-F	ccatgattacgaattcccgggcttcgacaattcaaatgtaagtcca
pCpt1a-LUC-R	tttggcgtcttccatgagctccaccatggtggcacaaatcgctatat

**ChIP**	
EcRE-F	aaatctactttgtttgggtcc
EcRE-R	aaggcgatgtttgcttgtgtg
KLF15BS1-F	actggccggcattgcccca
KLF15BS1-R	tgcatggggtgtcgttggt
KLF15BS2-F	actacaccccagtgccaca
KLF15BS2-R	gacaaacatagtcggcagt

Table S1. The list of all differentially modified proteins information.
